# Amblyostatin-1, the first salivary cystatin with host immunomodulatory and anti-inflammatory properties from the Neotropical tick *Amblyomma sculptum*, vector of Brazilian spotted fever

**DOI:** 10.3389/fimmu.2025.1585703

**Published:** 2025-07-17

**Authors:** Wilson Santos Molari, Mohamed Amine Jmel, Josiane Betim Assis, Alan Frazão-Silva, Júlia Moura Bernardi, Gretta Huamanrayme, José María Medina, Eliane Esteves, Solange Cristina Antão, Gabriel Cerqueira Alves Costa, Aparecida Sadae Tanaka, Andréa Cristina Fogaça, Zdenek Franta, Lucas Tirloni, Michalis Kotsyfakis, Anderson Sá-Nunes

**Affiliations:** ^1^ Department of Immunology, Institute of Biomedical Sciences, University of São Paulo, São Paulo, Brazil; ^2^ Institute of Parasitology, Biology Centre, Czech Academy of Sciences, České Budějovice, Czechia; ^3^ Université de Lorraine, CNRS, IMoPA, Nancy, France; ^4^ Departamento de Genética, Facultad de Ciencias, Universidad de Granada, Granada, Spain; ^5^ Department of Microbiology and Immunology, Frederick P. Whiddon College of Medicine, University of South Alabama, Mobile, AL, United States; ^6^ Department of Parasitology, Institute of Biomedical Sciences, University of São Paulo, São Paulo, SP, Brazil; ^7^ Department of Biochemistry, Federal University of São Paulo (UNIFESP), São Paulo, Brazil; ^8^ National Institute of Science and Technology in Molecular Entomology, National Council for Scientific and Technological Development, Rio de Janeiro, Brazil; ^9^ Department of Chemistry, Faculty of Science, University of South Bohemia, České Budějovice, Czechia; ^10^ Tick-Pathogen Transmission Unit, Laboratory of Bacteriology, Division of Intramural Research, National Institute of Allergy and Infectious Diseases, Hamilton, MT, United States; ^11^ Institute of Molecular Biology and Biotechnology, Foundation for Research and Technology-Hellas, Heraklion, Greece

**Keywords:** Amblyostatin-1, *Amblyomma sculptum*, immunomodulation, inflammation, tick-host interaction, tick saliva

## Abstract

**Introduction:**

The Neotropical tick *Amblyomma sculptum* is the primary vector of *Rickettsia rickettsii*, the causative agent of Brazilian spotted fever, a disease associated with high fatality rates. Tick saliva, a complex mixture of bioactive molecules essential for successful blood feeding, facilitates pathogen transmission and modulates host immune responses. A comprehensive evaluation of the salivary gland transcriptome database reveals that protease inhibitors are abundantly expressed molecules in tick saliva during feeding. Thus, this study aims to describe and characterize the most expressed member of the cystatin family identified in *Amblyomma sculptum* salivary transcriptome, named Amblyostatin-1.

**Methods:**

Bioinformatic tools were employed for *in silico* analysis of the Amblyostatin-1 sequence and structure. A recombinant version of Amblyostatin-1 was expressed in an *Escherichia coli* system, evaluated against a panel of cysteine proteases in biochemical assays, and used to generate antibodies in immunized mice. The biological activities of Amblyostatin-1 were assessed by its effects on dendritic cell maturation *in vitro* and in a carrageenan-induced inflammation model *in vivo*.

**Results:**

Based on its sequence and predicted three-dimensional structure, Amblyostatin-1 is classified as an I25B cystatin, and its recombinant form selectively inhibits cathepsins L, C, and S at different rates, with a low nanomolar *Ki* value of 0.697 ± 0.22 nM against cathepsin L. Regarding its biological activities, recombinant Amblyostatin-1 partially affects LPS-induced dendritic cell maturation by downmodulating the costimulatory molecules CD80 and CD86 at higher micromolar concentrations (3 µM) while promoting IL-10 production at nanomolar concentrations (100 nM). The apparent lack of Amblyostatin-1-specific antibody responses in immunized mice suggests an impairment of antigen processing and presentation *in vivo*. Furthermore, in a carrageenan-induced inflammation model, Amblyostatin-1 decreased edema formation and neutrophil infiltration into the skin without affecting other myeloid cells.

**Discussion:**

These findings establish Amblyostatin-1 as a novel salivary cystatin with immunomodulatory and anti-inflammatory properties, highlighting its potential as an immunobiological agent.

## Introduction

Blood feeding is vital for ticks to successfully develop and reproduce. However, during this process, they encounter barriers that protect the host’s integrity (1). Ticks from the Ixodidae family (hard ticks) must withstand vertebrate host defenses over several days to complete their feeding. To overcome this challenge, ticks have developed effective mechanisms throughout their evolution to avoid rejection during feeding (2). One such mechanism is the active secretion of saliva at the bite site, a fluid containing a complex mixture of molecules with anti-hemostatic and immunomodulatory properties. These molecules not only enable feeding but also facilitate the transmission of pathogens from the tick to the vertebrate host by modifying the host’s immune responses (3–5). In fact, ticks are vectors of wide range of pathogens, including *Rickettsia rickettsii* (Rocky Mountain/Brazilian spotted fever), *Borrelia burgdorferi* (Lyme disease), *Anaplasma phagocytophilum* (human granulocytic anaplasmosis), *Babesia microti* (babesiosis), and tick-borne encephalitis virus (tick-borne encephalitis), among others (6). *Amblyomma sculptum* [a member of the *Amblyomma cajennense* species complex (7)] is the most frequently reported tick species associated with human infestations in Brazil, where it serves as the main vector of *R. rickettsii* (8). Following transmission through the bite of an infected tick, the bacterium infects the host’s endothelial cells, leading to vasculitis, a condition that can progress to a potentially fatal outcome (9). Indeed, Brazil records high fatality rates associated with spotted fever each year (10).

Several host biological processes are modulated by tick salivary components, which include a diverse array of protease inhibitors found at the tick-host interface. These arthropod proteins target specific proteolytic enzymes involved in various physiological defense pathways, including coagulation, platelet aggregation, complement activation, inflammation, and adaptive immunity (11–13). Protease inhibitors represent the third most prevalent group of salivary molecules described in the TickSialoFam database, a comprehensive catalog of all available coding sequences derived from tick salivary gland transcriptomes (sialotranscriptomes) (14). The tick salivary protease inhibitors superfamily includes cystatins, Kazal- and Kunitz-type inhibitors, saposins, serpins, SPARC/Kazal proteins, tiropins, trypsin-like inhibitors (TIL), and carboxypeptidase inhibitors. Among these nine families, Kunitz-type inhibitors, serpins, and cystatins are the primary molecules secreted in tick saliva. These molecules are crucial for tick-host interactions and are vital for understanding the biological processes that govern this relationship (12, 15).

The cystatin family comprises a group of tight-binding inhibitors of host cysteine proteases that are known to be involved in multiple processes, including the regulation of proteolysis, antigen processing and presentation, development of immune system components, epidermal homeostasis, apoptosis, and neutrophil chemotaxis during inflammation (16). According to the MEROPS peptidase database (https://www.ebi.ac.uk/merops/), cystatins are part of the I25 family, which is further divided into four subfamilies (I25A-D) (17). Notably, only subfamilies I25A and I25B are present in ticks, with I25B cystatins identified in saliva (18). The first salivary cystatin characterized from ticks was Sialostatin L (19), whose RNA sequence was described in the original sialotranscriptome of *Ixodes scapularis* (20). Since then, salivary cystatins have been documented in several tick species, displaying a broad range of activities in vertebrate hosts and being explored as vaccine candidates and immunotherapeutic agents (reviewed by 13).

In the present study, we molecularly and functionally characterized Amblyostatin-1, the first salivary cystatin from the hard tick *A. sculptum*. The recombinant form of this protein shares the typical features of I25B cystatins exhibiting selective affinity for biologically significant cathepsins involved in dendritic cell (DC) biology and skin inflammation, along with low immunogenicity in the vertebrate host. Collectively, our findings suggest that Amblyostatin-1 has considerable potential for pharmacological applications, particularly due to its specificity arising from the evolutionary adaptations of arthropod salivary molecules at the tick-host interface that support the hematophagous lifecycle of these specific ectoparasites.

## Material and methods

### Animals

Male and female C57BL/6 mice, aged 6 to 12 weeks, were provided by the animal facility at the School of Medicine, University of Sao Paulo (FMUSP) and maintained at the animal facility of the Department of Immunology, Institute of Biomedical Sciences, University of Sao Paulo (ICB/USP). The animals were kept under specific-pathogen free conditions with food and water *ad libitum*.

All procedures involving vertebrate animals were conducted in accordance with Brazilian National Law number 11,794 (Arouca Law), Decree 6,899 and Normative Resolutions published by the National Council for the Control of Animal Experimentation (CONCEA), and were approved by the Institutional Animal Care and Use Committee (IACUC) from the University of Sao Paulo under the protocol number 4345130622.

### *In silico* analyses

The SignalP – 6.0 (21) tool was used to determine whether the amino acid sequence predicted in the *A. sculptum* sialotranscriptome contains a signal peptide. Putative glycosylation sites were assessed using the NetNGlyc – 1.0 (22) and NetOglyc – 4.0 (23) platforms. Additionally, the ProtParam Tool (24) was employed to evaluate theoretical parameters such as the isoelectric point, molecular weight and protein stability. Artificial intelligence-based modeling was performed using the AlphaFold 2 software (25), utilizing the amino acid sequence of the protein without the signal peptide. The resulting model was then uploaded to the GalaxyWEB server and subjected to further structural optimization using the GalaxyRefine tool (26, 27). Finally, the Galaxy refined model was submitted to the SAVES v6.0 platform for validation, where Ramachandran plot analysis, VERIFY 3D assessment, and ERRAT scoring were performed. The 3D structure was subsequently visualized using the PyMOL software (PyMOL, Molecular Graphics System, Version 2.5.5, Schrödinger, LLC).

To place Amblyostatin-1 and other *A. sculptum* cystatins within a phylogenetic context among tick cystatins, tick transcriptomic sequences were retrieved from various sources. Cystatin sequences were retrieved from the genome of *Amblyomma maculatum* (28), *Ixodes ricinus* (29) and *Ixodes scapularis* (30). Automatically annotated transcriptomic sequences were retrieved from the genomes of *Ixodes hexagonus*, *Ixodes pacificus*, and *Ixodes persulcatus*, through the Bioinformatics Platform for Agroecosystem Arthropods (https://bipaa.genouest.org).

BLAST was used to align all retrieved sequences against known sequences in the SwissProt-UniProt database (31, 32). Protein sequences showing significant similarity to known cystatins were selected. Additionally, InterProScan was used to identify which of the retrieved sequences contained cystatin domains (33). This information was combined to generate a catalog of cystatin proteins from *A. sculptum*, *A. maculatum* and five different *Ixodidae* species.

To investigate the similarities and conserved regions of the cystatins, a multiple sequence alignment was performed using Clustal Omega (34). Spurious sequences and misaligned regions were removed using trimAl (35) and the phylogenetic tree was generated using ggtree, an R package for visualization of tree-like structures (36).

### Expression of recombinant Amblyostatin-1

The coding sequence of Amblyostatin-1 (GenBank accession number PV164378), excluding the signal peptide, was synthetized and cloned into a pET-17b expression vector (Gene Universal Inc., Newark, DE, USA), which was used to transform BL21 (DE3) pLysS *Escherichia coli* cells (Invitrogen, Carlsbad, CA, USA). Subsequently, a pre-culture was prepared using a positive clone, which was then transferred to 6 L of LB medium containing ampicillin (100 μg/mL) and chloramphenicol (34 μg/mL) and incubated at 30°C with shaking at 225 rpm. Culture growth was monitored by measuring the optical density (O.D.) at 600 nm. Once the culture reached the optimal growth rate for initiating expression (established at 0.6 < O.D. < 0.8), isopropyl-beta-D-thiogalactoside (IPTG) was added to a final concentration of 1 mM to induce protein expression. Cell pellets were resuspended in Tris 20 mM, kept on ice, sonicated, and washed, yielding insoluble fraction containing inclusion bodies. The resulting inclusion bodies were dissolved in 25 mL of guanidine buffer (50 mM Tris, 5 mM DTT, and 7.5 M guanidine) for 2 hours at room temperature, and the insoluble material was removed by centrifugation at 15,000 *g*/30 min/4°C. Recombinant Amblyostatin-1 was then refolded by diluting the solution 50-fold in a buffer consisting of 0.1 M TBS, supplemented with 1 M guanidine HCl, 500 mM L-arginine, and 10% glycerol. Following overnight incubation, the resulting solution was concentrated by filtration through a 3 kDa MWCO membrane (Amicon® Centrifugal Filter Unit, Sigma Aldrich, Darmstadt, Germany) to achieve the desired volume. Purification was performed by size exclusion chromatography using a Superdex 75 column (Sigma Aldrich, Darmstadt, Germany) coupled to an ÄKTA PureTM instrument (Life Sciences, Piscataway, NJ, Germany), equilibrated in 100 mM Tris, 140 mM NaCl, 3 mM KCl, 500 mM L-arginine, 1 M guanidine, 10% glycerol, pH 7.5, and the resulting protein was stored at 4°C until use.

Additional attempts to express Amblyostatin-1 using the HEK293 mammalian cell system (VR2001 vector), the *Drosophila* S2 expression system (pMT/bip/v5-his A vector), and the *Pichia pastoris* expression system (pPICZαC vector) were unsuccessful (data not shown). However, the expression of the recombinant protein in BL21 (DE3) pLysS *Escherichia coli* cells and the refolding protocol used were previously validated for other tick cystatins (19, 37–40).

### Enzymatic activity assay

Cystatins are well-described competitive inhibitors of cysteine proteases that bind to the active site of proteases, preventing peptide bond cleavage and acting as pseudo-substrates (41). To evaluate the inhibition specificity, five cathepsins were chosen (L, S, C, B, and H), whose Km and Vmax are well established and can be referenced in the BRaNE database (https://brenda-enzymes.org/). Briefly, 1 μM of recombinant Amblyostatin-1 was preincubated with the selected cathepsins at the concentrations indicated in the **Supplementary Table S1** for 10 minutes at 23° C. Following, 250 μM substrate was added, and the formation of the reaction product was monitored at 365/450 nm (excitation/emission) using an Infinite 200 PRO 96-well plate fluorescence reader (Tecan, Männedorf, Switzerland) to assess the loss of activity in the presence of the inhibitor.

Enzymatic assays for cathepsin L were conducted in the presence of Amblyostatin-1, using a methodology previously described (42). Briefly, active cathepsin L (6 nM) was incubated with varying concentrations of Amblyostatin-1 in 50 mM sodium acetate buffer (pH 5.5) at 37°C for 10 minutes. The fluorogenic substrate *Z*-Phe-Arg-AMC was then added (43), and fluorescence was monitored at 380/460 nm (excitation/emission) using a Synergy HT microplate reader (BioTek Instruments Inc., Winooski, VT, USA). Fluorescence readings were taken at 30°C over a 15 minutes period, and enzyme activity was estimated by their Vmax. Residual activity was calculated as Vmax of enzyme activity in the presence of the inhibitor divided by the Vmax of the control enzyme (without inhibitor). Residual Vmax values were plotted, and the dissociation constant (*K_i_*) was calculated through nonlinear regression analysis using the Morrison equation for tight-binding inhibition (44).

### DC cultures

Bone marrow cells were harvested from the femurs of mice, and erythrocytes were lysed using ACK lysing buffer (Gibco; Thermo Fisher Scientific, Grand Island, NY, USA). Suspensions containing 3 × 10^5^ cells/mL were prepared in complete medium [RPMI 1640, supplemented with GlutaMAX, 25 mM HEPES, 10% fetal bovine serum (FBS), 100 units/mL penicillin, 100 μg/mL streptomycin and 5.5 × 10–^5^ M 2-mercaptoethanol – all Gibco products (Thermo Fisher Scientific, Grand Island, NY, USA)] and distributed in 100 mm diameter Petri dishes (10 mL). The cells were incubated with 20 ng/mL of murine GM-CSF at 37 °C and 5% CO_2_ to induce DC differentiation, as previously described (45, 46). Briefly, after 4 days of incubation, half of the culture volume was replaced with fresh complete medium containing 40 ng/mL murine GM-CSF. Following a total of 7 days of incubation, nonadherent cells were collected and 2 × 10^5^ cells/well in 100 μL were distributed into flat bottom 96-well plates. These cells were preincubated for 1 hour at 37° C and 5% CO_2_ with either complete medium alone or medium containing different concentrations of recombinant Amblyostatin-1 (0.1 to 3 μM), followed by stimulation with 200 ng/mL of ultrapure LPS (InvivoGen, San Diego, CA, USA) for 24 hours. Unstimulated controls (cells maintained in medium only) were also included. DCs were then collected and prepared for flow cytometry analysis, while the supernatant was stored at -80°C for subsequent cytokine evaluation.

### Mice immunization

Mice were immunized subcutaneously with Amblyostatin-1 (5 µg/animal) emulsified in Alum (Reheis, Berkeley Heights, NJ, USA) as an adjuvant, while control group received only PBS emulsified in Alum, as previously described (47).The immunization process was repeated twice at two-week intervals, resulting in a total of three immunizations. Two weeks later, an intravenous booster (0.1 µg/animal) was administered, and three days later, blood was collected via submandibular vein puncture, after which serum was separated for further experiments. As an internal control, another group of mice was immunized under the same conditions with AsKunitz, a previously described Kunitz-type inhibitor (48).

### ELISA

Cytokines present in the DC culture supernatant were detected by ELISA. IL-12p40 and TNF-α levels were determined using the BD OptEIA ELISA Set kit (BD Biosciences Pharmingen, San Diego, CA, USA), while IL-6 and IL-10 levels were measured using the ELISA Max™ kit (Biolegend, San Diego, CA, USA), according to the manufacturers’ instructions. The cytokine concentrations for each sample were calculated based on standard curves and expressed in pg/mL using GraphPad Prism version 8.0.2 (GraphPad Software, Boston, Massachusetts USA). The limits of detection were 15.6 pg/mL for IL-12p40 and TNF-α, 7.80 pg/mL for IL-6, and 31.3 pg/mL for IL-10.

In another set of experiments, we performed an in-house ELISA to detect serum IgG antibodies, adapted from previous work of our group (47). Briefly, plates were coated with Amblyostatin-1, AsKunitz, or *A. sculptum* saliva, blocked with PBS containing 10% FBS, and incubated with a pool of serum (1:1000) from control mice or mice immunized with Amblyostatin-1 or AsKunitz. Bound antibodies were detected using HRP-conjugated goat anti-mouse IgG and revealed with TMB substrate (both from Invitrogen, Rockford, lL, USA). The absorbance was measured at 450 nm and data was presented as the optical density (OD) of the readings.

### Gel electrophoresis and Western blot

To assess the presence and specificity of antibodies in the serum of immunized animals, *A. sculptum* saliva (250 μg), recombinant Amblyostatin-1 (1 μg) and recombinant AsKunitz (1 μg) were subjected to electrophoresis separation using Bolt™ 4-12% Bis-Tris Plus gels (Invitrogen) under the following conditions: 200 V, 120 mA, and 25 W for 30 minutes. Following electrophoresis, the first gel was stained with Coomassie blue dye for one hour and subsequently washed overnight with water to remove unbound dye. The second gel was used for Western blot analysis, as previously described (49). Briefly, the proteins were transferred onto a nitrocellulose membrane using the iBlot^®^ Dry Blotting System (Invitrogen). The membranes were blocked with a Tris buffer containing 10% FBS for two hours at room temperature. After blocking, the membranes were washed with Tris Buffer containing 0.05% Tween-20 (TBST) and incubated overnight at 4°C with pooled serum from non-immunized mice or mice immunized with Amblyostatin-1 or AsKunitz (both at 1:1000 dilution). The membranes were then washed and incubated for one hour with horseradish peroxidase-conjugated goat anti-mouse IgG antibodies (Invitrogen) for detection. Protein bands were visualized using the Novex^®^ Chemiluminescent Substrate Reagent Kit (Invitrogen) and captured using a gel documentation system (ImageQuant™ LAS 500, GE Healthcare Bio-Sciences AB, Uppsala, Sweden).

### Carrageenan-induced paw inflammation

The carrageenan-induced paw inflammation model was employed to test the potential anti-inflammatory effects of Amblyostatin-1 *in vivo*. For this purpose, mice received 25 µL of a 1% λ-carrageenan solution (Sigma-Aldrich, St Louis, MO, USA) in PBS, with or without Amblyostatin-1 (0.1 µM) administered into the plantar pad of the hind paws. A control group received only PBS. Paw thickness was assessed using a micrometer prior to inoculation and again at 1, 4, and 24 hours post-inoculation, with edema defined by the difference in thickness compared to the initial measurement. Four- and 24-hours post-inoculation, the skin was excised to analyze *in vivo* cellular infiltration. Briefly, the paw skin was collected with the aid of a scalpel, cut into small fragments, weighed, and subjected to digestion in a solution of collagenase (1 mg/mL) and DNAse (0.5 mg/mL) for 40 minutes at 37°C with agitation at 1250 rpm. Subsequently, samples were filtered through a 40 μm cell strainer (Corning, Durham, NC, USA), washed with PBS, and transferred to 12 × 75 mm polypropylene tubes (BD Falcon, Franklin Lakes, NJ, USA) for flow cytometry analysis.

### Flow cytometry

DCs from the previously described cultures were collected, washed with PBS, and incubated for 10 minutes at 4°C with Live/Dead-Aqua to assess cell viability and with anti-CD16/CD32 antibodies to block Fc receptors. After washing, the cells were incubated with a mixture of the following fluorochrome-conjugated monoclonal antibodies (all from BD Biosciences): anti-CD11b-APC-Cy7 (clone M1/70), anti-CD11c-APC (clone N418), anti-CD40-PE (clone 3/23), anti-CD80-FITC (clone 16-10A1), anti-CD86-PECy7 (clone GL-1), anti-F4/80-Pacific Blue (clone BMS), anti-MHC II-PerCp-Cy5.5 (clone M5/114.15.2) for 30 minutes at 4°C. Following staining, the cells were washed and resuspended in cytometry buffer (PBS containing 2% FBS) and kept on ice until acquisition. The gating strategy is outlined in **Supplementary Figure S1**.

To analyze the cell infiltrate in carrageenan-induced edema, the paw skin cells were labeled with Live/Dead-AmCyan for 10 minutes at 4°C. After incubation, the cells were washed and labeled with a mixture of fluorochrome-conjugated monoclonal antibodies against Ly6G-FITC (clone 1A8), CD11b-APC-Cy7 (clone M1/70), CD45-APC (clone 30-F11) diluted in flow cytometry buffer for 30 minutes at 4°C. Following staining, the cells were washed and resuspended in cytometry buffer and kept on ice until acquisition. The gating strategy is outlined in the **Supplementary Figure S2**.

In both cases, samples were acquired using the FACSCanto™ II flow cytometer (BD Biosciences, San José, CA, USA), and the events were analyzed using FlowJo software, version 10.0.7 (Tree Star, Ashland, OR, USA).

### Statistical analysis

Quantitative data were expressed as the mean ± standard error of the mean (SEM). The sample size for each set of experiments is depicted in the legend of the figures. Experimental groups were compared with their respective controls using one-way ANOVA followed by Tukey post-test using GraphPad Prism version 8.0.2 (GraphPad Software). Differences considered statistically significant at *p* ≤ 0.05, and the exact *p* value is indicated in each figure.

## Results

### Amblyostatin-1 is a typical member of I25B cystatin subfamily

**Figure 1:**
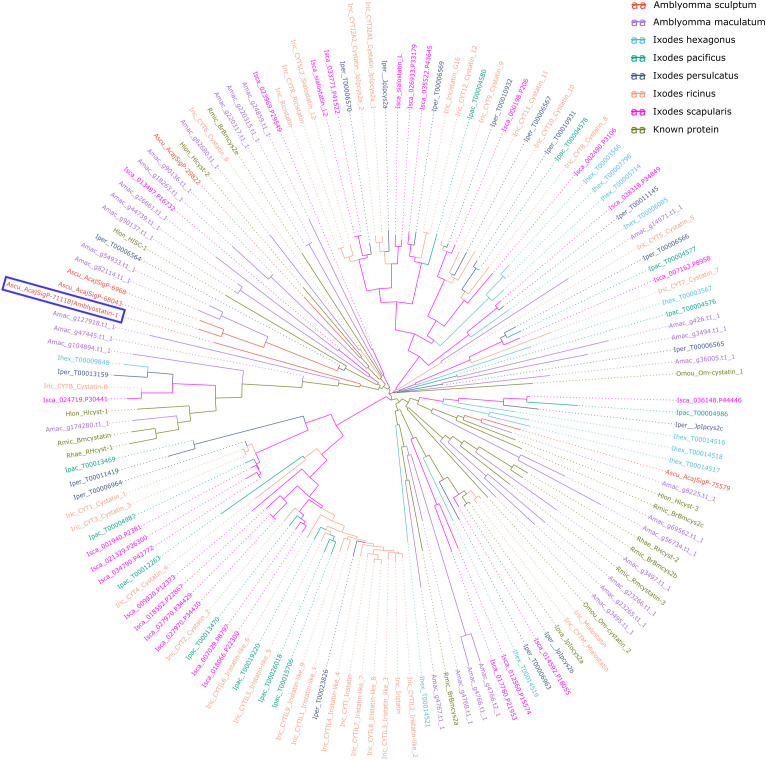
Evolutionary relationships of *A. sculptum* cystatins among tick cystatins. The phylogenetic tree, generated using ggtree package, displays cystatin sequences from *A. sculptum* (red), *A. maculatum*, five *Ixodidae* species, and previously characterized cystatins. Different species are distinguished by color and by the initial part of the protein ID, where the first letter indicates the genus, followed by the first three letters of the species name. Amblyostatin-1 is highlighted within a blue lined box.

**Figure 2:**
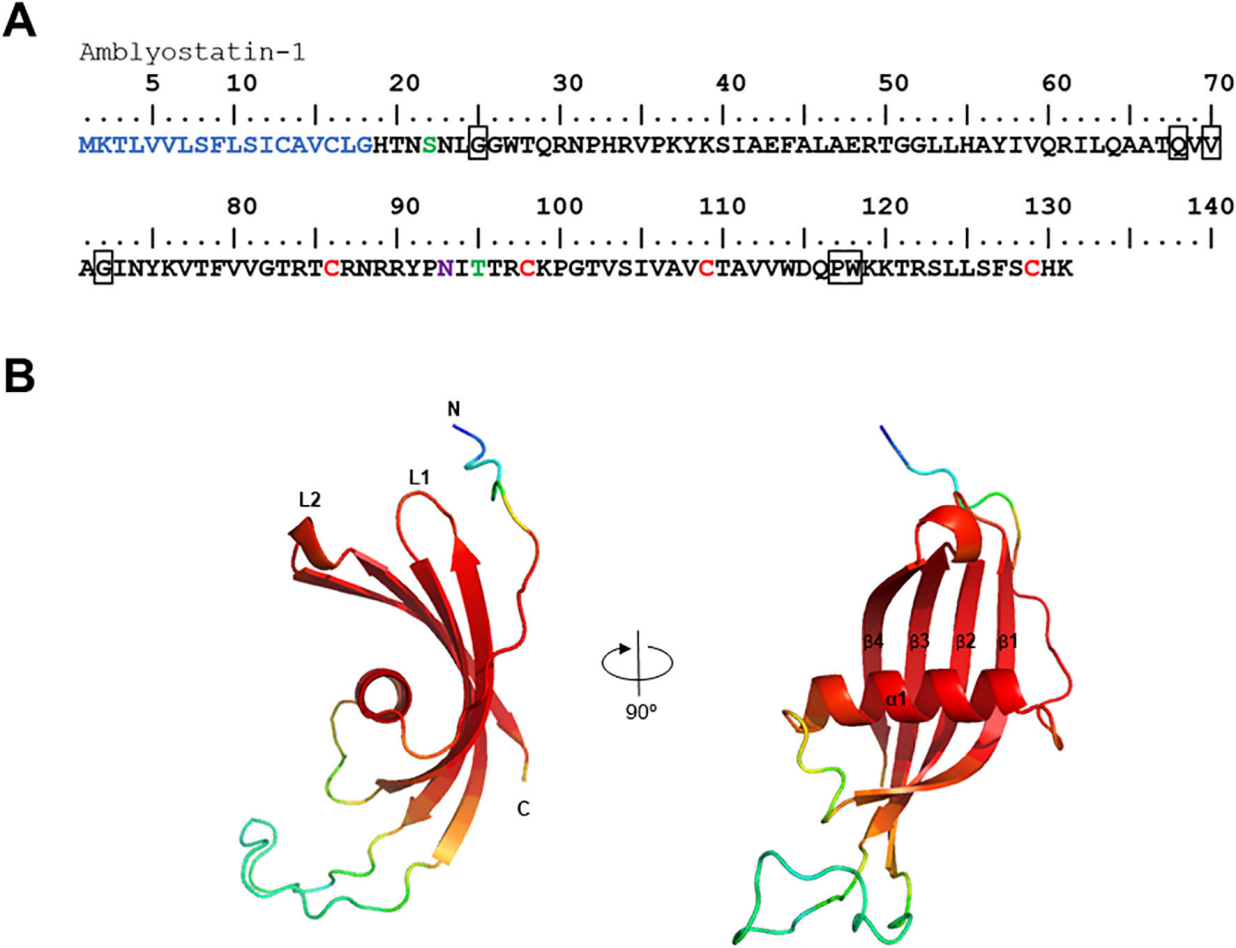
*In silico* analysis of the Amblyostatin-1. **(A)** Amino acid sequence of Amblyostatin-1. The signal peptide is shown in blue, cysteine residues in red, O-glycosylation sites in green, and N-glycosylation sites in purple. The boxes highlight the three cystatin motifs: (G), (QxVxG), and (PW). **(B)** Three-dimensional structure modeled using AlphaFold 2 software. Colors indicate the confidence score of the model: red – very high (pLDDT > 90), light red – high (90 > pLDDT > 70), yellow – low (70 > pLDDT > 50), blue – very low (pLDDT < 50).

Our group has published a comparative sialotranscriptome analysis of unfed and partially fed *Amblyomma sculptum* ticks (50). In that study, six cystatin sequences were identified (**Table 1**), five of which were predicted to be secreted in saliva based on SignalP analysis. The phylogenetic analysis (**Figure 1**) revealed that the specific secreted *A. sculptum* cystatins exhibit sequence divergence when compared to cystatins of *A. maculatum*, *Ixodes* spp., and cystatins characterized in previous research by many different groups globally (15). This prompted us to perform a functional characterization of the transcript AcajSIGP-71118 (denoted with a box in **Figure 1**), which was the most transcriptionally upregulated cystatin gene in partially fed females (**Table 1**). Its 393 base pair sequence encodes for a 131 amino acid protein with a predicted molecular weight of 12,710.75 Da and an isoelectric point of 10.59. The first 18 amino acids in the N-terminus correspond to the signal peptide, and the protein contains four cysteine residues, one potential N-glycosylation site, and two possible O-glycosylation sites (**Figure 2A**). The protein was named Amblyostatin-1, because it is the first characterized salivary cystatin from *A. sculptum*.

**Table 1:** Coding sequences (CDSs) of cystatins differentially expressed in the salivary glands of *A. sculptum* in response to feeding.

CDS	Annotation	Accession number (GenBank)	Fold-change (RNA-seq)	
			Unfed	Fed (72 hours)
AcajSigP-71118	tick_cistatins_1 - signalP detected	PV164378	0.06	16.38
AcajSigP-68043	tick salivary cystatin - signalP detected	PV164380	0.11	9.05
AcajSigP-6968	tick salivary cystatin - signalP detected	PV164379	0.26	3.81
AcajSigP-75579	cystatin - signalP detected	JAU02687.1	0.50	1.95
Acaj-76693	intracellular cystatin	JAU02694.1	1.07	0.93
AcajSigP-29822	tick_cistatins_1 - signalP detected	PV164377	3.91	0.25

Data extracted from *A. scultum* sialotranscriptome (50).

Amblyostatin-1 sequence presents the three typical cystatin motifs – N-terminal G, QxVxG and a C-terminal PW segment – also found in other cystatins (**Figure 2B**). The three-dimensional structure of the secreted form of Amblyostatin-1 (excluding signal peptide) was developed using the AlphaFold 2 software. The predicted structure reveals a twisted antiparallel β-sheet composed of four β-strands connected by two loops (L1 and L2) surrounding an α-helix, which is characteristic of cystatins. The loop region and N-terminus are likely interaction sites for Amblyostatin-1 with its targets (**Figure 2B**) The validation of the refined model shows high structural quality: ERRAT2 yielded an overall quality factor of 95.455 (**Supplementary Figure S3A**), while the Ramachandran plot indicated that 93.9% of residues fall within the most favored regions (**Supplementary Figure S3B**), indicating excellent backbone conformational accuracy. The Verify3D assessment revealed that 54.87% of residues achieved a 3D–1D score of at least 0.1 (**Supplementary Figure S3C**), suggesting a moderate level of compatibility between the predicted fold and its amino acid sequence. Similar results were obtained with other structure prediction platforms that use homology modeling, such as Modeller and Rosetta (data not shown). Overall, these findings confirm that the *in silico* modeling of Amblyostatin-1 structure exhibits robust stereochemical integrity and moderate sequence–structure compatibility.

### Recombinant Amblyostatin-1 targets cathepsin L, S, and C

**Figure 3:**
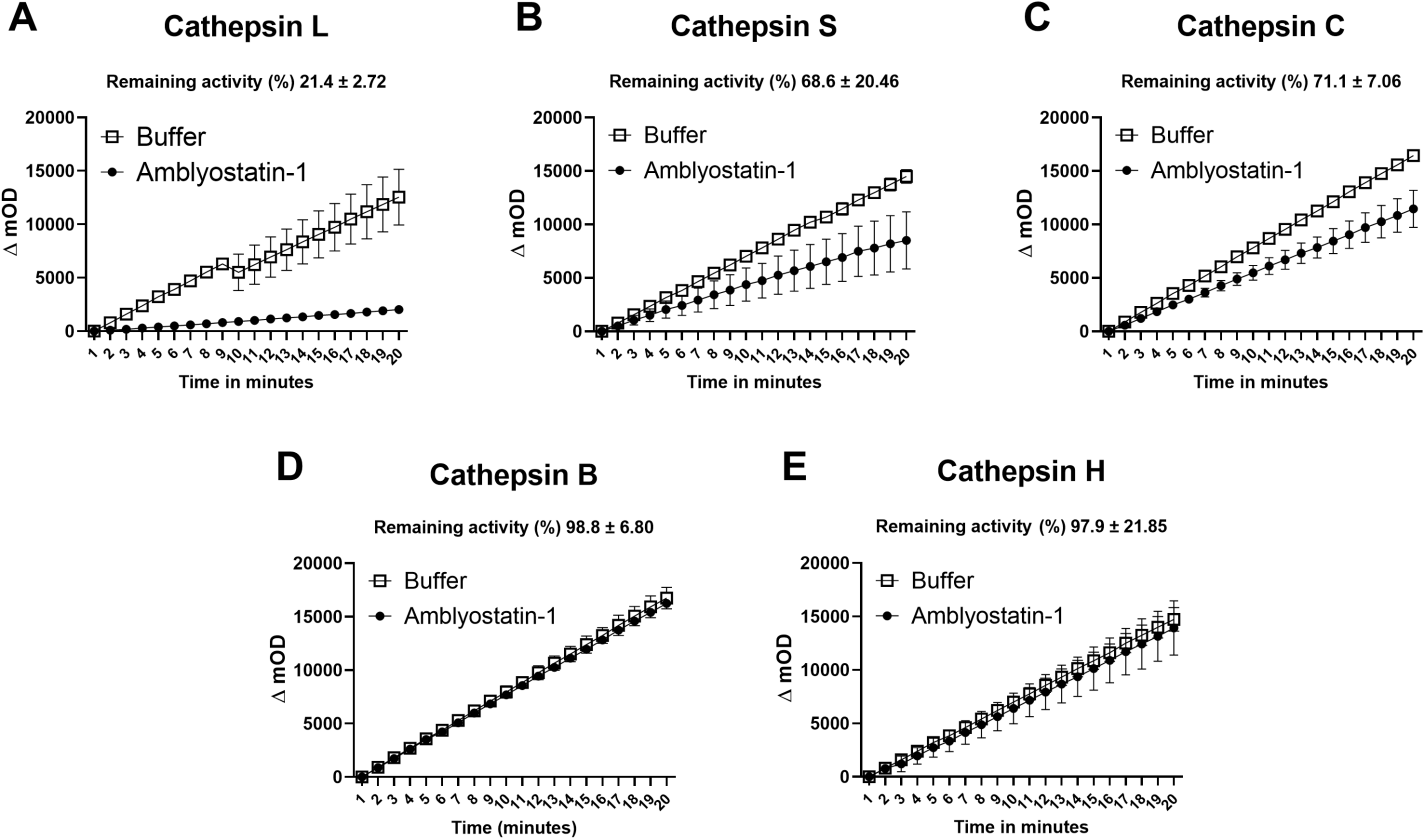
Residual enzymatic activity of different cathepsins in the presence of Amblyostatin-1. The kinetics of cystatin inhibitory activity were evaluated against cathepsin L **(A)**, S **(B)**, C **(C)**, B **(D)** and H **(E)**. Inhibition percentages for each enzyme were calculated by comparing the initial rates of enzymatic activity in the absence and presence of Amblyostatin-1, under the conditions described in the **Supplementary Table S1**.

The activity of recombinant Amblyostatin-1 as a cysteine protease inhibitor was tested against five different cathepsins. Inhibition percentages for each enzyme were calculated by comparing the initial rates of enzymatic activity in the absence and presence of Amblyostatin-1. Under the conditions described in the **Supplementary Table S1**, Amblyostatin-1 inhibits cathepsin L (**Figure 3A**), cathepsin S (**Figure 3B**), and cathepsin C (**Figure 3C**), while no inhibitory effect was observed on cathepsin B (**Figure 3D**) or cathepsin H (**Figure 3E**).

Due to limited protein yield, an enzymatic kinetic assay with varying concentrations of Amblyostatin-1 was performed only in the presence of cathepsin L, the protease that exhibited the highest apparent inhibition profile (**Figure 3A**). A significant inhibition of cathepsin L protease activity was observed, with an estimate *Ki* value of 0.697 ± 0.22 nM (**Supplementary Figure S4**).

### Amblyostatin-1 selectively modulates DC biology

Given that salivary cystatins from other tick species with similar target profiles interfere with DC phenotypes, we tested whether Amblyostatin-1 would affect the maturation of these cells induced by a bacterial mimic. When cultured with Amblyostatin-1 alone, the basal expression of costimulatory/accessory molecules CD40, CD80, and CD86 remained unchanged. Upon stimulation with LPS from gram-negative bacteria, all these molecules were upregulated as expected. However, when DCs were preincubated with Amblyostatin-1 prior to LPS stimulation, a selective concentration-response inhibition was observed.

**Figure 4:**
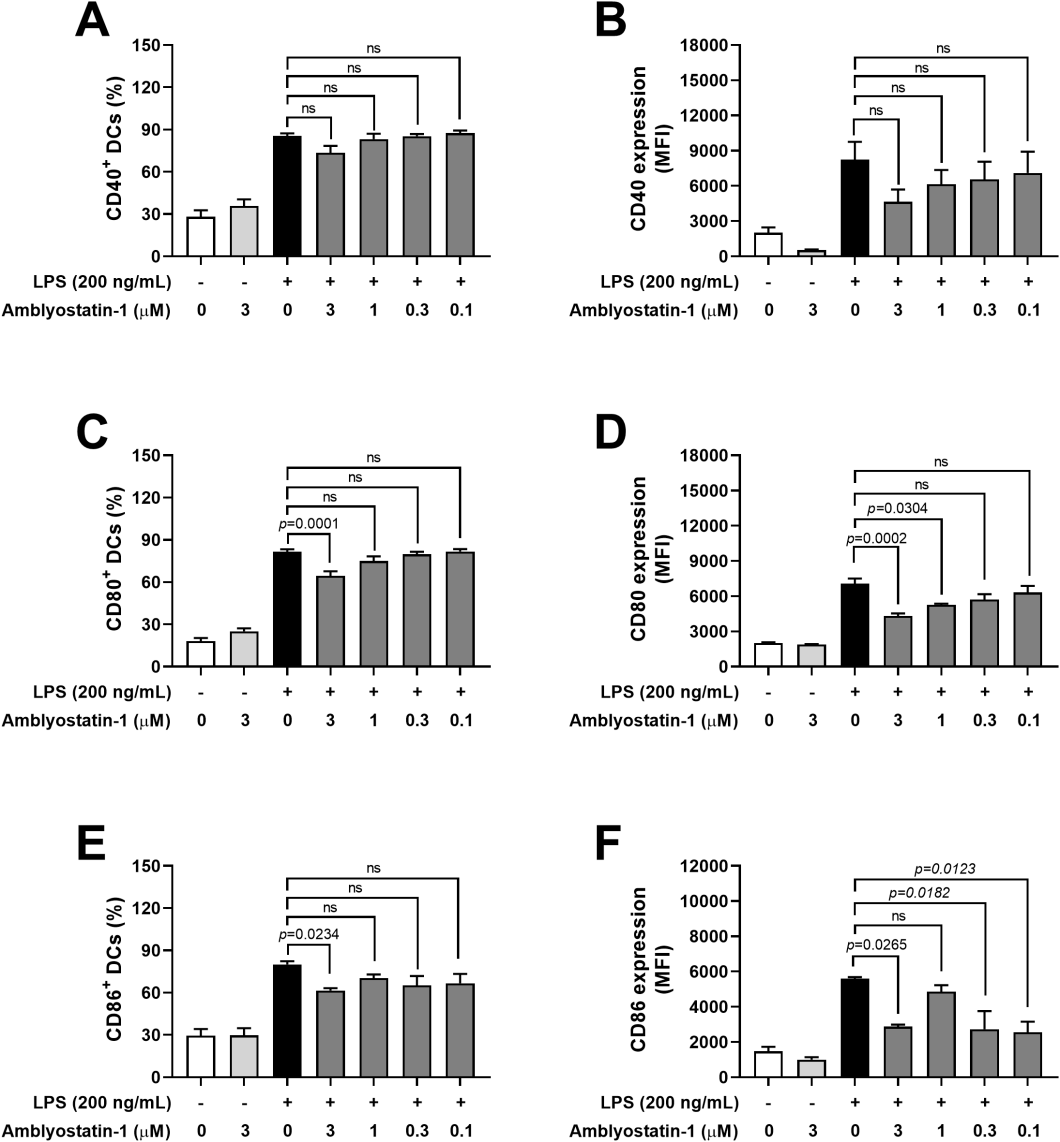
Expression of accessory/costimulatory molecules by DCs in the presence of Amblyostatin-1. DCs were preincubated with medium or Amblyostatin-1 at different concentrations for 1 hour and subsequently stimulated with LPS for 24 hours. DCs were evaluated by flow cytometry for the percentage of CD40^+^ **(A)**, CD80^+^ **(C)**, and CD86^+^ **(E)** cells and for the median fluorescence intensity (MFI) of CD40 **(B)**, CD80 **(D)**, and CD86 **(F)**. Data represent the mean ± SEM in the different groups from two independent experiments. Significant *p* values are indicated in the figure. All comparisons were made by one-way ANOVA with Tukey post-test (n = 4-9 per group). ns, non-significant.

A slight decrease in the percentage of CD40^+^ DCs and the median fluorescence intensity (MFI) of this marker was noted in the presence of 3 μM Amblyostatin-1, although the difference did not reach statistical significance (**Figures 4A, B**, respectively). Conversely, preincubation of DCs with 3 μM Amblyostatin-1 followed by LPS stimulation significantly reduced the percentage of CD80^+^ and CD86^+^ DCs (**Figures 4C, E**, respectively). For MFI of these markers, reductions were observed at 3 and 1 μM of Amblyostatin-1 for CD80 (**Figure 4D**) and at 3, 0.3 and 0.1 μM for CD86^+^ (**Figure 4F**).

**Figure 5:**
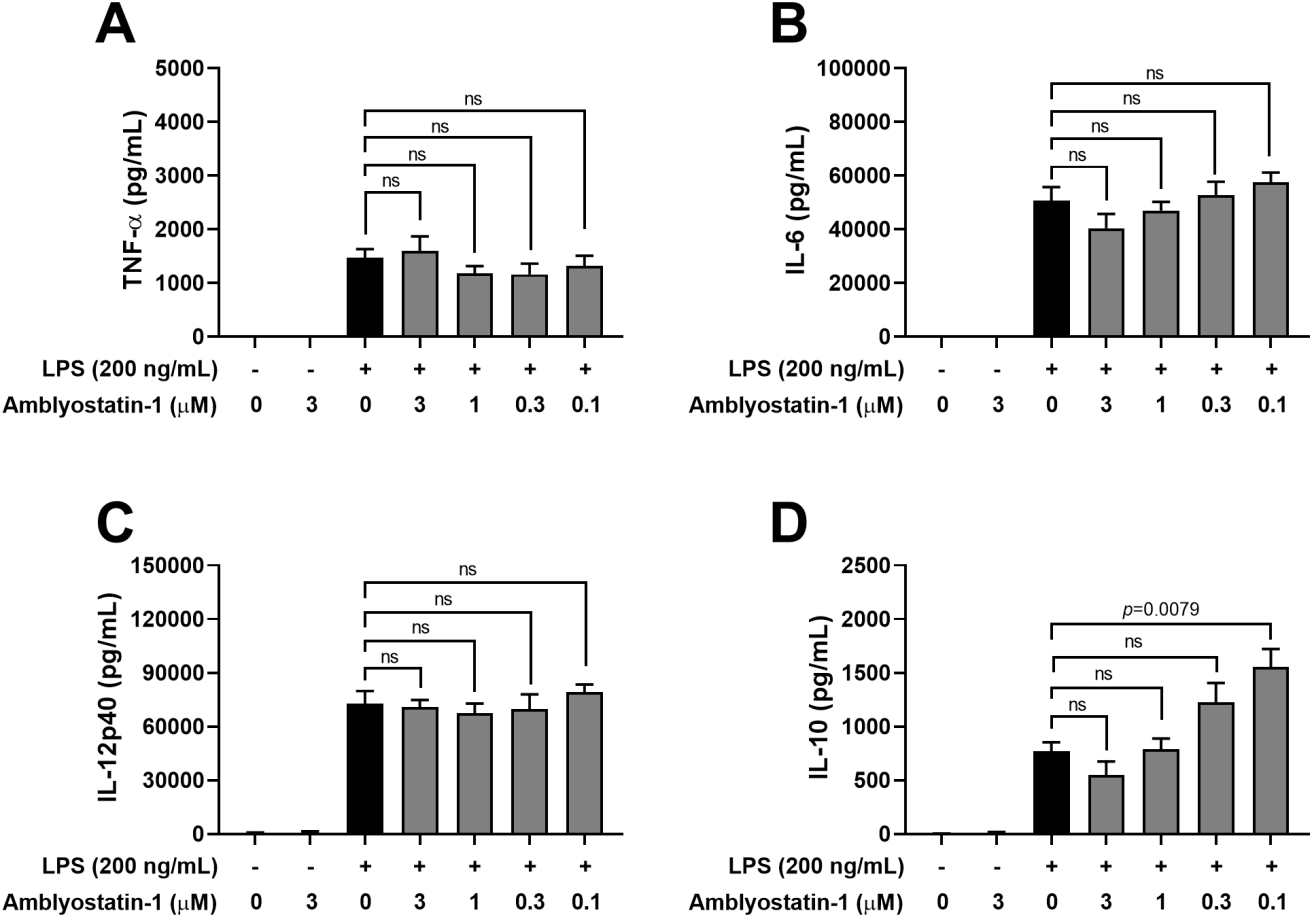
Cytokine production by DCs in the presence of Amblyostatin-1. DCs were preincubated with medium or Amblyostatin-1 at different concentrations for 1 hour and subsequently stimulated with LPS for 24 hours. Culture supernatant was used to evaluate TNF-α **(A)**, IL-6 **(B)**, IL-12p40 **(C)** and IL-10 **(D)** levels. Data represent the mean ± SEM in the different groups from two independent experiments. Significant *p* values are indicated in the figure. All comparisons were made by one-way ANOVA with Tukey post-test (n = 4 per group). ns, non-significant.

Next, the production of cytokines by DCs was assessed in the culture supernatants. Similar to DCs maintained in medium only, cells incubated with Amblyostatin-1 produced very low or undetectable levels of all evaluated cytokines. As expected, DCs stimulated with LPS produced high levels of TNF-α (**Figure 5A**), IL-6 (**Figure 5B**) and IL-12p40 (**Figure 5C**), and IL-10 (**Figure 5D**), as is typical for activated cells. Preincubation of DCs with Amblyostatin-1 did not alter the production of TNF-α, IL-6 or IL-12p40 at any tested concentrations. Interestingly, an inverse concentration-response increase in IL-10 production was observed in LPS-stimulated DCs preincubated with the inhibitor, reaching statistical significance at 0.1 μM Amblyostatin-1 (**Figure 5D**). The viability of DCs under various conditions was also evaluated to rule out any cytotoxic effects of Amblyostatin-1, and cell viability was found to be similar across all experimental groups (data not shown).

### Recombinant Amblyostatin-1 does not induce antibody generation in mice

**Figure 6:**
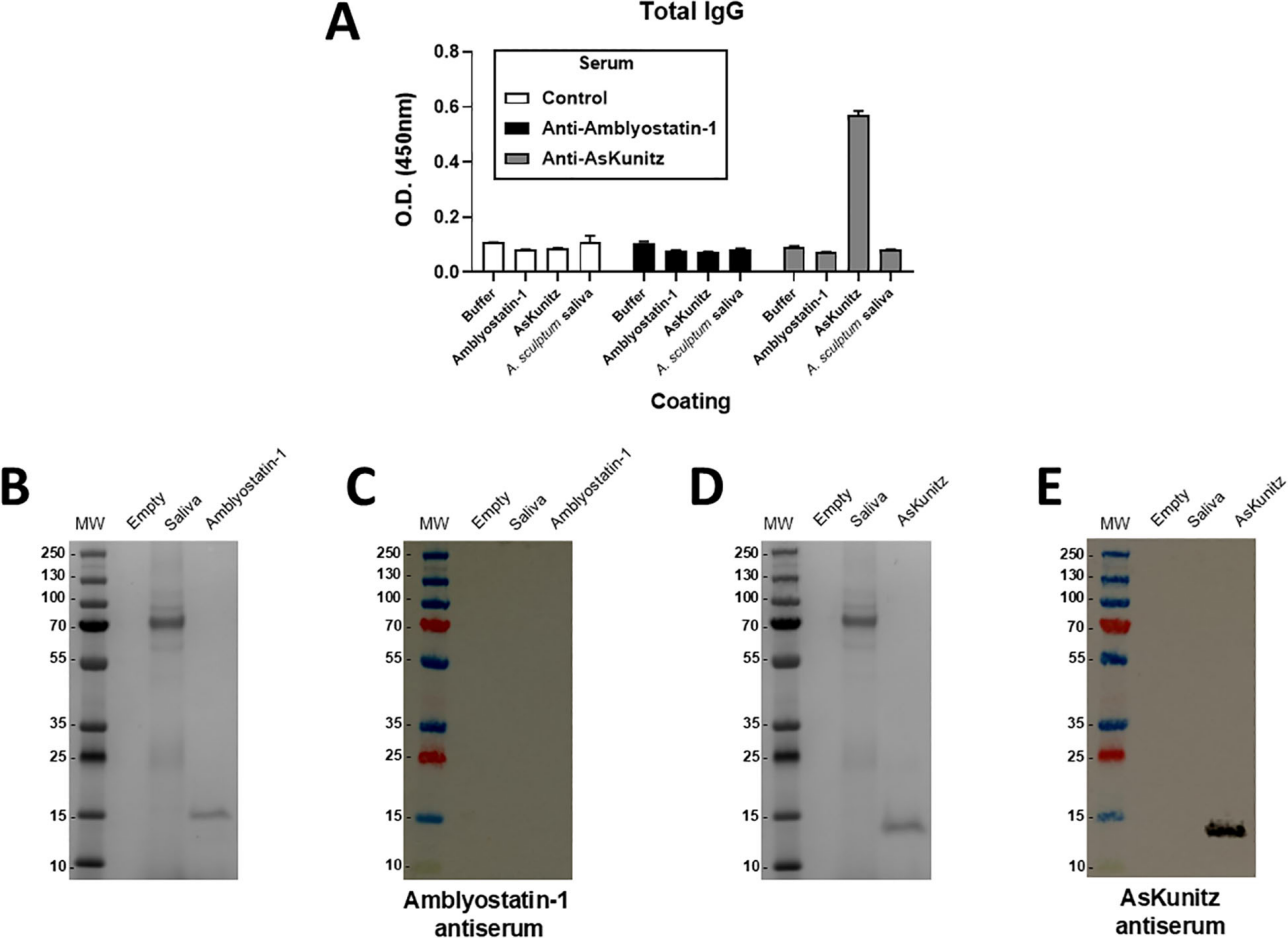
Amblyostatin-1 does not induce humoral immune responses in immunized mice. Mice were immunized with recombinant Amblyostatin-1 or AsKunitz as described in Material and Methods. Control mice received only PBS in adjuvant. Blood of these mice was collected and serum was separated for the assays. **(A)** ELISA assay to evaluate the antiserum recognition of Amblyostatin-1, AsKunitz, and *A. sculptum* saliva, used as a plate coating (n = 4 animals per group); **(B)** Gel electrophoresis of *A. sculptum* saliva and recombinant Amblyotatin-1; **(C)** Western blot assay to evaluate the antiserum recognition of Amblyostatin-1 and *A. sculptum* saliva; **(D)** Gel electrophoresis of *A. sculptum* saliva and recombinant AsKunitz; **(E)** Western blot assay to evaluate the antiserum recognition of Amblyostatin-1 and *A. sculptum* saliva.

The presence and specificity of anti-Amblyostatin-1 antibodies in immunized mice was assessed. As a reference, another group of mice was immunized with AsKunitz, a Kunitz-type inhibitor previously described (48). As expected, serum from animals that received the adjuvant only (control) did not recognize recombinant Amblyostatin-1, AsKunitz, or *A. sculptum* saliva (**Figure 6A**). Intriguingly, the serum of mice immunized with recombinant Amblyostatin-1 did not recognize either the respective inhibitor or *A. sculptum* saliva, similar to the control. In contrast, the serum of mice immunized with recombinant AsKunitz recognized the respective inhibitor; however, *A. sculptum* saliva was not recognized (**Figure 6A**). To evaluate the specificity of these interactions, the sera were blotted against the respective targets. Multiple bands for *A. sculptum* saliva and a single band for recombinant Amblyostatin-1 were observed in gel electrophoresis, close to the 15 kDa marker (**Figure 6B**). However, no reaction was detected when Amblyostatin-1 antiserum was blotted against the same material (**Figure 6C**). To rule out any experimental artifacts, a similar approach was applied to AsKunitz, where multiple bands for *A. sculptum* saliva and a single band for recombinant AsKunitz were observed in gel electrophoresis, between the 10 and 15 kDa markers (**Figure 6D**). A strong reaction was observed when AsKunitz antiserum was blotted against recombinant AsKunitz, but not against saliva (**Figure 6E**), suggesting that this Kunitz-type inhibitor is either not secreted in *A. sculptum* saliva or its amounts are below the detection limit of the assay. Together, these findings indicate that Amblyostatin-1 presents low immunogenicity to mice.

### Amblyostatin-1 reduces edema and leukocyte infiltration to the skin

**Figure 7:**
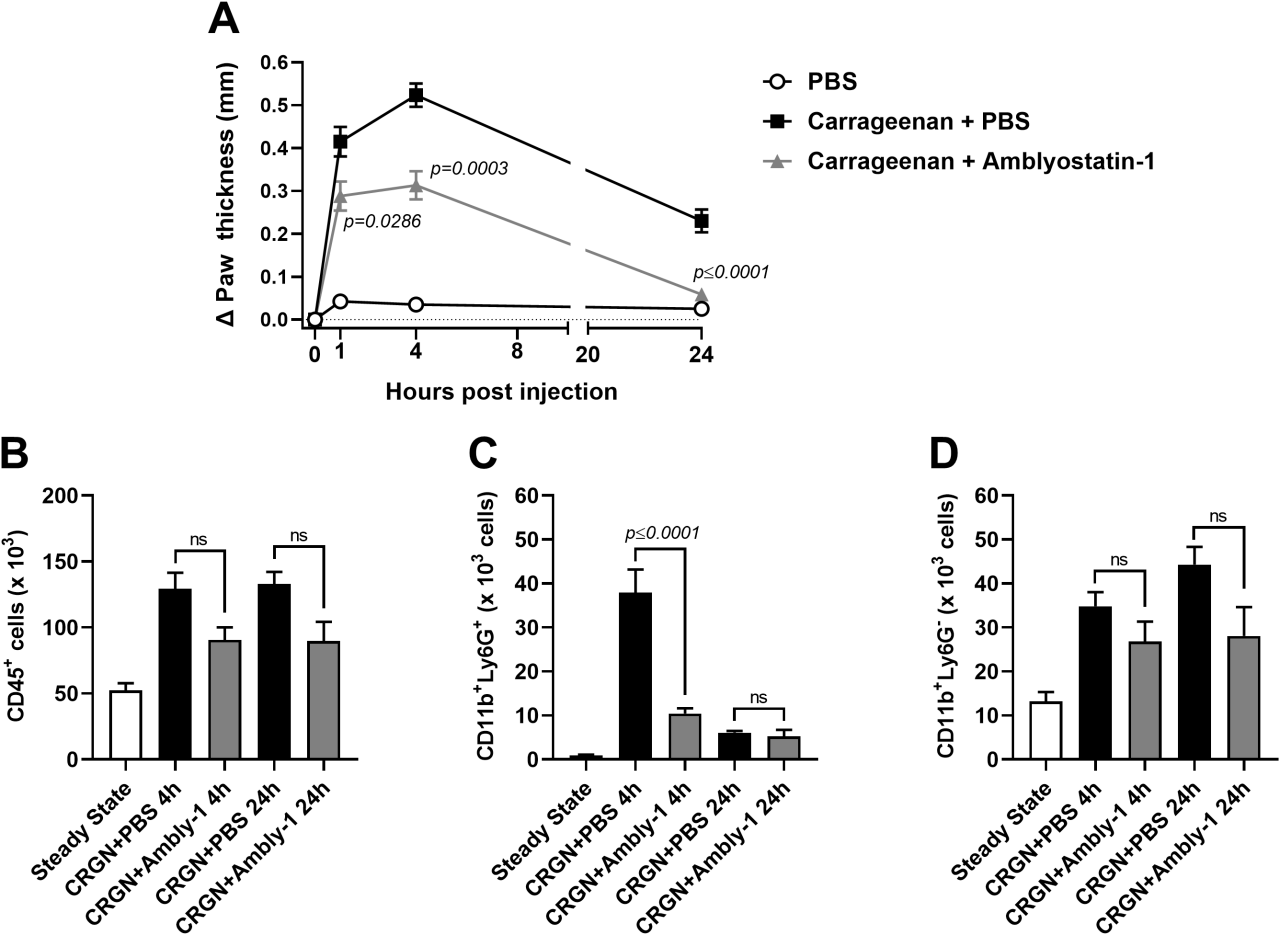
Amblyostatin-1 presents anti-inflammatory activity *in vivo*. Mice received an inoculation of carrageenan (1%) plus PBS or carrageenan (1%) plus Amblyostatin-1 (0.1 μM) in their hind paw, and the edema formation was evaluated at 1, 4 and 24 hours post-inoculation **(A)**. In another set of experiments, the skin was removed, processed and the inflammatory infiltrate was evaluated by flow cytometry. The following populations were phenotyped: **(B)** total immune cells (CD45^+^ cells), **(C)** neutrophils (CD11b^+^Ly6G^+^ cells), and **(D)** non-neutrophil myeloid cells (CD11b^+^Ly6G^-^ cells). Data represent the mean ± SEM in the different groups from two independent experiments. Significant *p* values are indicated in the figure. All comparisons were made by one-way ANOVA with Tukey post-test (n = 4-6 animals per group). CRGN, carrageenan; Ambly-1, Amblyostatin-1; ns, non-significant.

The enzymatic profile and increased production of IL-10 point out a potential anti-inflammatory role for Amblyostatin-1. To test this hypothesis, the carrageenan-induced paw edema – an *in vivo* experimental model of acute skin inflammation – was performed in the presence or absence of the inhibitor. Remarkably, Amblyostatin-1 exhibited substantial anti-inflammatory activity, resulting in reduced paw edema throughout the evaluation period, with statistically significant effects observed at 4 and 24 hours of post-carrageenan inoculation (**Figure 7A**).

The skin cellular infiltrate was characterized at these time points to identify the populations affected by Amblyostatin-1. Animals inoculated with carrageenan plus PBS exhibited an increased number of immune cells (CD45^+^ cells) at both 4 and 24 hours post-injection compared to the steady-state group. A 30% reduction in CD45^+^ cells was observed in the group co-inoculated with carrageenan plus Amblyostatin-1 at both time points; however, this difference was not statistically significant when compared to the carrageenan plus PBS group (**Figure 7B**). As expected, there was an acute peak of neutrophils (CD11b^+^Ly6G^+^ cells) at 4 hours post-carrageenan inoculation, which is typical for this model. However, in the presence of Amblyostatin-1, neutrophil infiltration to the skin was significantly reduced. No differences between the groups were observed at 24 hours post-inoculation (**Figure 7C**). For the other myeloid cell populations (CD11b^+^Ly6G^-^ cells), a similar trend to that of CD45^+^ cells was observed, showing a non-significant reduction in the group receiving carrageenan plus Amblyostatin-1 at 4 and 24 hours post-inoculation (23% and 36%, respectively) when compared to the group receiving carrageenan plus PBS (**Figure 7D**).

It is important to emphasize that the tissue digestion process utilizing collagenase and DNAse may be aggressive to the cells, potentially affecting cell viability. Nevertheless, all the experimental groups were similarly impacted by this procedure (data not shown), supporting the conclusion that the observed phenotype is due to the effects of Amblyostatin-1 on cell migration and/or recruitment, rather than an experimental artifact.

## Discussion

This study provides structural, phylogenetic, biochemical, and functional insights into Amblyostatin-1, a novel member of the cystatin family from *A. sculptum* saliva. The Amblyostatin-1 transcript (AcajSIGP-71118), originally described in the species’ sialotranscriptome, was shown to be upregulated in partially engorged females compared to non-fed females (50), suggesting its involvement in blood feeding. *In silico* analysis of the protein’s physicochemical properties, along with the modeled three-dimensional structure, classifies Amblyostatin-1 as a member of the I25B subfamily of protease inhibitors (17). Notably, despite being uncommon among these cystatins, Amblyostatin-1 has two predicted O-glycosylation sites and one N-glycosylation site, indicating potential post-translational modifications that may significantly impact the protein functionality (23). If these predicted modifications occur, they could result in structural alterations or enhanced functions of the protein, influencing its folding, stability, and interactions, thereby impacting its biological activity.

Among all tested expression systems, only the prokaryotic system successfully yielded a sufficient amount of protein for biochemical and biological assays (data not shown). Importantly, the inhibition profile against cathepsins matches those documented for different cystatins, indicating that the functional conformation of the recombinant protein may be preserved. In fact, in our experience, recombinant cystatins expressed in both prokaryotic and eukaryotic systems exhibit comparable affinity constants against target cysteine proteases (19, 51–53). Nevertheless, heterologous expression of tick salivary cystatins in prokaryotic systems has led to the purification of proteins with inhibitory activities in the picomolar range (19, 37), and the inhibition profile of Amblyostatin-1 agrees with that of most tick salivary cystatins previously reported (40).

Although various cystatin transcripts have been sequenced in *A. sculptum* sialotranscriptome (50), sequence similarity is variable, reflecting the diverse functions of these molecules (54, 55). Besides the three typical cystatin motifs (N-terminal G, QxVxG and a C-terminal PW segment), Amblyostatin-1 shares conserved regions with selected cystatins that align with the cystatin alpha-helix and the region following the QxVxG motif, forming the first loop (hairpin loop 1). The similarity among these proteins primarily occurs in the alpha-helix forming region. Functional cystatins inhibit papain-like cysteine proteases (family C1) through a reversible binding mechanism, competing with substrates for the enzyme’s active site (56, 57). A proposed inhibitor-enzyme interaction model suggests that the cystatin binding site is complementary to the protease’s active-site cleft, enabling effective inhibition via a tight-binding mechanism (i.e., *Ki* in the nanomolar to picomolar range) (57, 58). The inhibition of cathepsin L by Amblyostatin-1 was evaluated under the assumption that it follows a mechanism similar to that of previously described cystatins. Kinetic analysis using the Morrison equation, which characterizes the reversible inhibition of enzyme-catalyzed reactions by tight-binding inhibitors (44). confirmed a low nanomolar *Ki* value for cathepsin L inhibition.

Given that two previously described salivary cystatins from ticks – OmC2 from *O. moubata* and Sialostatin L from *I. scapularis* – also target cathepsin S and have been shown to inhibit DC maturation (59, 60), Amblyostatin-1 was tested for this activity. Because DCs are the most powerful skin-resident antigen-presenting cells and readily interact with salivary molecules and other physiological tick components during infestation, tick salivary molecules present an exquisite propensity to modulate DC biology (61). Indeed, Amblyostatin-1 was found to affect DC maturation by reducing the expression of costimulatory molecules CD80 and CD86 following LPS stimulation, without significantly changing the expression of CD40, or the production of proinflammatory cytokines, or the viability of these cells. It is known that inhibition of cathepsin S directly impacts the processing of the major histocompatibility complex class II (MHC II)-associated invariant chain in DCs (62, 63). This may indicate that Amblyostatin-1 interferes with the maturation and function of these cells. Although we have not tested whether Amblyostatin-1 is internalized by DCs to exert its biological activities, this capacity has been demonstrated for other tick-derived cystatins. For example, OmC2 was taken up by human DCs and translocated to proteolytically active compartments involved in antigen processing, where it bound to cathepsins S and C (59, 60). Furthermore, murine DCs incubated with Sialostatin L accumulate an invariant chain intermediate (Ii-p10), which is cleaved into the class II-associated invariant chain peptide (CLIP) within lysosomal compartments, a process dependent on cathepsin S (59, 60). A similar mechanism may occur with Amblyostatin-1, potentially explaining the absence of T-dependent antibodies following immunization with the protein, as their production is reliant on antigen processing and presentation by both DCs and B lymphocytes. Moreover, the unknown concentration of Amblyostatin-1 in tick saliva, along with the modulation of its expression during different phases of infestation, may limit the development of detectable antibody responses under natural conditions, though this assumption requires further investigation. Indeed, tick salivary cystatins have previously been proposed as “silent antigens” because they are not recognized upon natural exposure of vertebrate hosts to ticks, and humoral immune recognition requires the injection of artificially high amounts of recombinant proteins (64).

Unlike other tick salivary cystatins, Amblyostatin-1 increased IL-10 production by LPS-stimulated DCs at nanomolar concentrations. Interestingly, the whole saliva of a range of tick species also induces the IL-10 production by activated DCs; however, this phenotype has been attributed to the presence of non-protein salivary molecules such as prostaglandin E_2_ (PGE_2_) and adenosine (65, 66). IL-10 is an anti-inflammatory and regulatory cytokine, part of a cytokine family that plays multiple roles in health and disease (67, 68). Although this phenotype is novel for tick salivary cystatins, scientific literature shows that helminth cystatins can induce IL-10 production and promote the development and influx of IL-10-producing cells across various experimental models. Notable examples include AvCystatin/Av17 from *Acanthocheilonema viteae* (69–71), Onchocystatin from *Onchocerca volvulus* (72), CsStefin-1 from *Clonorchis* sinensis (73), *Sj-*Cys from *Schistosoma japonicum* (74), Ts-Cys from *Trichinella* sp*iralis* (75), among others. As these cystatins belong to the I25A and I25B subfamilies and exhibit multiple target specificities, it is challenging to establish a unified pattern that explains IL-10 induction by Amblyostatin-1. Therefore, findings regarding Amblyostatin-1 as an additional salivary molecule capable of enhancing IL-10 production in activated DCs warrant further investigation. For instance, kinetic analyses and isothermal titration calorimetry may be used to better characterize the biochemical properties of the molecule and strengthen these findings. In addition, *in vivo* studies using RNA interference-mediated silencing of Amblyostatin-1 in ticks during infestation would validate its functional role in modulating host immune responses.

Together, the induction of IL-10 and the inhibition of cathepsins involved in cell migration and function pointed out for a potential modulation of inflammatory responses by Amblyostatin-1. Indeed, the recombinant protein has been shown to reduce edema formation in a carrageenan-induced inflammation model, as well as decrease neutrophil infiltration into the skin. Other salivary cystatins with similar target profiles that have demonstrated comparable anti-inflammatory effects by inhibiting neutrophil migration *in vivo* include Sialostatin L, Sialostatin L2, Iristatin, Ricistatin, and Mialostatin (19, 37, 38, 40, 76). Although this model does not replicate the tick-host skin interface, it is a skin inflammation protocol known to recruit neutrophils, the primary cell type that migrates during initial tick infestations and may play a role in the formation of feeding lesion (77–80).

It is important to highlight that, contrary to ticks found in the Northern Hemisphere, very little is known about the saliva of endemic tick species in South America. To date, only five *A. sculptum* salivary proteins have been characterized at a functional level: Amblyomin-X and AsKunitz, both of which are both Kunitz-type protease inhibitors (48, 81, 82); evasin ACA-01, a chemokine binding protein (83); As8.9kDa and AsBasicTail, representing the 8.9 kDa and basic tail families, respectively (48). In addition, PGE_2_ (a non-protein molecule) has been isolated from *A. sculptum* saliva (46). Thus, Amblyostatin-1 emerges as a novel immunomodulator from *A. sculptum*, contributing to the expanding list of tick salivary cystatins that exert immunomodulatory roles on the immune system of the vertebrate host.

In conclusion, Amblyostatin-1 belongs to I25B subfamily of protease inhibitors and functions as a regulator of DC activation. It downmodulates the expression of costimulatory molecules CD80 and CD86, while selectively enhancing IL-10 production by LPS-stimulated DCs, and does not impact TNF-α, IL-12p40, or IL-6 production. Additionally, Amblyostatin-1 reduces inflammation in a carrageenan-induced paw edema murine model and affects neutrophil infiltration without interfering with the migration of other leukocyte types at the site of inflammation. The inhibitory kinetics of Amblyostatin-1 toward its other targets still remain to be determined to confirm whether the affinities for particular cathepsins are associated with the phenotypes observed. These findings underscore the potential use of these molecules in developing strategies to control tick parasitism, as well as their potential application as therapeutic agents for human inflammatory and autoimmune diseases, particularly those involving pathogenic protease activity.

## Data Availability

The datasets presented in this study can be found in online repositories. The names of the repository/repositories and accession number(s) can be found below: https://www.ncbi.nlm.nih.gov/genbank/, PV164378 https://www.ncbi.nlm.nih.gov/genbank/, PV164380 https://www.ncbi.nlm.nih.gov/genbank/, PV164379 https://www.ncbi.nlm.nih.gov/genbank/, JAU02687.1 https://www.ncbi.nlm.nih.gov/genbank/, JAU02694.1 https://www.ncbi.nlm.nih.gov/genbank/, PV164377.

## References

[1] Nuttall PA. Tick saliva and its role in pathogen transmission. Wien Klin Wochenschr. (2019) 1(135):165–176. doi: 10.1007/s00508-019-1500-y, PMID: 31062185 PMC10118219

[2] Kitsou C Fikrig E Pal U. Tick host immunity: vector immunomodulation and acquired tick resistance. Trends Immunol. (2021) 42:554–574. doi: 10.1016/j.it.2021.05.005, PMID: 34074602 PMC10089699

[3] Francischetti IM Sá-Nunes A Mans BJ Santos IM Ribeiro JM. The role of saliva in tick feeding. Front Biosci (Landmark Ed). (2009) 14:2051–88. doi: 10.2741/3363, PMID: 19273185 PMC2785505

[4] Kotál J Langhansová H Lieskovská J Andersen JF Francischetti IM Chavakis T. Modulation of host immunity by tick saliva. J Proteomics. (2015) 128:58–68. doi: 10.1016/j.jprot.2015.07.005, PMID: 26189360 PMC4619117

[5] Šimo L Kazimirova M Richardson J Bonnet SI. The essential role of tick salivary glands and saliva in tick feeding and pathogen transmission. Front Cell Infect Microbiol. (2017) 7:281. doi: 10.3389/fcimb.2017.00281, PMID: 28690983 PMC5479950

[6] Piesman J Eisen L. Prevention of tick-borne diseases. Annu Rev Entomol. (2008) 53:323–43. doi: 10.1146/annurev.ento.53.103106.093429, PMID: 17877457

[7] Nava S Beati L Labruna MB Cáceres AG Mangold AJ Guglielmone AA. Reassessment of the taxonomic status of *Amblyomma cajennense* () with the description of three new species, *Amblyomma tonelliae* n. sp., *Amblyomma interandinum* n. sp. and *Amblyomma patinoi* n. sp., and reinstatement of *Amblyomma mixtum*, and *Amblyomma sculptum* (Ixodida: Ixodidae). Ticks Tick Borne Dis. (2014) 5:252–76. doi: 10.1016/j.ttbdis.2013.11.004, PMID: 24556273

[8] Nogueira BCF Campos AK Muñoz-Leal S Pinter A Martins TF. Soft and hard ticks (Parasitiformes: ixodida) on humans: A review of Brazilian biomes and the impact of environmental change. Acta Trop. (2022) 234:P. 106598. doi: 10.1016/j.actatropica.2022.106598, PMID: 35841953

[9] Chen LF Sexton DJ. What’s new in rocky mountain spotted fever? Infect Dis Clin North Am. (2008) 22:415–32. doi: 10.1016/j.idc.2008.03.008, PMID: 18755382

[10] Ministério da Saúde. Óbitos confirmados de Febre Maculosa. Brasil, Regiões e Unidades Federadas (Infecção) – 2007 a 2025. Saúde de A a Z, Brasília, DF (sede do Ministério da Saúde) (2025). Available online at: https://www.gov.br/saude/pt-br/assuntos/saude-de-a-a-z/f/febre-maculosa/situacao-epidemiologica/obitos-de-febre-maculosa-brasil-grandes-regioes-e-unidades-federadas-infeccao-2007-a-2025/view (Accessed May 15 2025).

[11] Martins LA Kotál J Bensaoud C Chmelař Kotsyfakis M. Small protease inhibitors in tick saliva and salivary glands and their role in tick-host-pathogen interactions. Biochim Biophys Acta Proteins Proteom. (2020) 1868:P. 140336. doi: 10.1016/j.bbapap.2019.140336, PMID: 31816416

[12] Jmel MA Voet H Araújo RN Tirloni L Sá-Nunes A Kotsyfakis M. Tick salivary Kunitz-type inhibitors: targeting host hemostasis and immunity to mediate successful blood feeding. Int J Mol Sci. (2023) 24. doi: 10.3390/ijms24021556, PMID: 36675071 PMC9865953

[13] Černý J Arora G. Proteases and protease inhibitors in saliva of hard ticks: biological role and pharmacological potential. Adv Parasitol. (2024) 126:229–251. doi: 10.1016/bs.apar.2024.09.001, PMID: 39448192

[14] Ribeiro JMC Mans BJ. TickSialoFam (TSFam): A database that helps to classify tick salivary proteins, A review on tick salivary protein function and evolution, with considerations on the tick sialome switching phenomenon. Front Cell Infect Microbiol. (2020) 10:P. 374. doi: 10.3389/fcimb.2020.00374, PMID: 32850476 PMC7396615

[15] Chmelař J Kotál J Langhansová H Kotsyfakis M. Protease inhibitors in tick saliva: the role of serpins and cystatins in tick-host-pathogen interaction. Front Cell Infect Microbiol. (2017) 7:P. 216. doi: 10.3389/fcimb.2017.00216, PMID: 28611951 PMC5447049

[16] Parizi LF Sabadin GA Alzugaray MF Seixas A Logullo C Konnai S. *Rhipicephalus microplus* and *Ixodes ovatus* cystatins in tick blood digestion and evasion of host immune response. Parasit Vectors. (2015) 8:P. 122. doi: 10.1186/s13071-015-0743-3, PMID: 25889092 PMC4340882

[17] Rawlings ND Barrett AJ Thomas PD Huang X Bateman A Finn RD. The merops database of proteolytic enzymes, their substrates and inhibitors in 2017 and a comparison with peptidases in the panther database. Nucleic Acids Res. (2018) 46:D624–32. doi: 10.1093/nar/gkx1134, PMID: 29145643 PMC5753285

[18] Schwarz A Valdés JJ Kotsyfakis M. The role of cystatins in tick physiology and blood feeding. Ticks Tick Borne Dis. (2012) 3:117–27. doi: 10.1016/j.ttbdis.2012.03.004, PMID: 22647711 PMC3412902

[19] Kotsyfakis M Sá-Nunes A Francischetti IM Mather TN Andersen JF Ribeiro JM. Antiinflammatory and immunosuppressive activity of Sialostatin L, A salivary cystatin from the tick *Ixodes scapularis* . J Biol Chem. (2006) 281:26298–307. doi: 10.1074/jbc.M513010200, PMID: 16772304

[20] Valenzuela JG Francischetti IM Pham VM Garfield MK Mather TN Ribeiro JM. Exploring the sialome of the tick *Ixodes scapularis* . J Exp Biol. (2002) 205:2843–64. doi: 10.1242/jeb.205.18.2843, PMID: 12177149

[21] Teufel F Almagro Armenteros JJ Johansen AR Gíslason MH Pihl SI Tsirigos KD. Signalp 6.0 predicts all five types of signal peptides using protein language models. Nat Biotechnol. (2022) 40:1023–1025. doi: 10.1038/s41587-021-01156-3, PMID: 34980915 PMC9287161

[22] Gupta R Brunak S. Prediction of glycosylation across the human proteome and the correlation to protein function. Pac Symp Biocomput. (2002) 7:310–22., PMID: 11928486

[23] Steentoft C Vakhrushev SY Joshi HJ Kong Y Vester-Christensen MB Schjoldager KT. Precision mapping of the human O-galnac glycoproteome through simplecell technology. EMBO J. (2013) 32:1478–88. doi: 10.1038/emboj.2013.79, PMID: 23584533 PMC3655468

[24] Gasteiger E Hoogland C Gattiker A Duvaud S Wilkins MR Appel RD. Protein identification and analysis tools on the expasy server. In: The Proteomics Protocols Handbook. Humana Press, Totowa, Nj (2005). p. 571–607., PMID:

[25] Jumper J Evans R Pritzel A Green T Figurnov M Ronneberger O. Highly accurate protein structure prediction with alphafold. Nature. (2021) 596:583–589. doi: 10.1038/s41586-021-03819-2, PMID: 34265844 PMC8371605

[26] Heo L Park H Seok C. Galaxyrefine: protein structure refinement driven by side-chain repacking. Nucleic Acids Res. (2013) 41:W384–8. doi: 10.1093/nar/gkt458, PMID: 23737448 PMC3692086

[27] Lee GR Heo L Seok C. Effective protein model structure refinement by loop modeling and overall relaxation. Proteins. (2016) 84 Suppl 1:293–301. doi: 10.1002/prot.24858, PMID: 26172288

[28] Ribeiro JMC Bayona-Vásquez NJ Budachetri K Kumar D Frederick JC Tahir F. A draft of the genome of the gulf coast tick, *Amblyomma maculatum* . Ticks Tick Borne Dis. (2023) 14:P. 102090. doi: 10.1016/j.ttbdis.2022.102090, PMID: 36446165 PMC9898150

[29] Cerqueira De Araujo A Noel B Bretaudeau A Labadie K Boudet M Tadrent N. Genome sequences of four *Ixodes* species expands understanding of tick evolution. BMC Biol. (2025) 23:P. 17. doi: 10.1186/s12915-025-02121-1, PMID: 39838418 PMC11752866

[30] De S Kingan SB Kitsou C Portik DM Foor SD Frederick JC. A high-quality *Ixodes scapularis* genome advances tick science. Nat Genet. (2023) 55:301–311. doi: 10.1038/s41588-022-01275-w, PMID: 36658436

[31] Altschul SF Gish W Miller W Myers EW Lipman DJ. Basic local alignment search tool. J Mol Biol. (1990) 215:403–10. doi: 10.1016/S0022-2836(05)80360-2, PMID: 2231712

[32] Consortium U. Uniprot: the universal protein knowledgebase in 2023. Nucleic Acids Res. (2023) 51:D523–31. doi: 10.1093/nar/gkac1052, PMID: 36408920 PMC9825514

[33] Jones P Binns D Chang HY Fraser M Li W McAnulla C. Interproscan 5: genome-scale protein function classification. Bioinformatics. (2014) 30:1236–40. doi: 10.1093/bioinformatics/btu031, PMID: 24451626 PMC3998142

[34] Madeira F Madhusoodanan N Lee JE Eusebi A Niewielska A Tivey A. The embl-ebi job dispatcher sequence analysis tools framework in 2024. Nucleic Acids Res. (2024) 52:W521–5. doi: 10.1093/nar/gkae241, PMID: 38597606 PMC11223882

[35] Capella-Gutiérrez S Silla-Martínez JM Gabaldón T. Trimal: A tool for automated alignment trimming in large-scale phylogenetic analyses. Bioinformatics. (2009) 25:1972–3. doi: 10.1093/bioinformatics/btp348, PMID: 19505945 PMC2712344

[36] Yu G. Using ggtree to visualize data on tree-like structures. Curr Protoc Bioinf. (2020) 69:E96. doi: 10.1002/cpbi.96, PMID: 32162851

[37] Kotsyfakis M Karim S Andersen JF Mather TN Ribeiro JM. Selective cysteine protease inhibition contributes to blood-feeding success of the tick *Ixodes scapularis* . J Biol Chem. (2007) 282:29256–63. doi: 10.1074/jbc.M703143200, PMID: 17698852

[38] Kotál J Stergiou N Buša M Chlastáková A Beránková Z Řezáčová P. The structure and function of iristatin, a novel immunosuppressive tick salivary cystatin. Cell Mol Life Sci. (2019) 76:2003–2013. doi: 10.1007/s00018-019-03034-3, PMID: 30747251 PMC11105445

[39] Kotál J Buša M Urbanová V Řezáčová P Chmelař J Langhansová H. Mialostatin, a novel midgut cystatin from *Ixodes ricinus* ticks: Crystal structure and regulation of host blood digestion. Int J Mol Sci. (2021) 22. doi: 10.3390/ijms22105371, PMID: 34065290 PMC8161381

[40] Martins LA Buša M Chlastáková A Kotál J Beránková Z Stergiou N. Protease-bound structure of ricistatin provides insights into the mechanism of action of tick salivary cystatins in the vertebrate host. Cell Mol Life Sci. (2023) 80:P. 339. doi: 10.1007/s00018-023-04993-4, PMID: 37898573 PMC11071917

[41] Turk V Bode W. The cystatins: protein inhibitors of cysteine proteinases. FEBS Lett. (1991) 285:213–9. doi: 10.1016/0014-5793(91)80804-C, PMID: 1855589

[42] Costa GCA Torquato RJS de Morais Gomes V Rosa-Fernandes L Palmisano G Tanaka AS. Functional characterization of a cystatin A from the bat Myotis davidii. Comp Biochem Physiol B Biochem Mol Biol. (2024) 274:111003. doi: 10.1016/j.cbpb.2024.111003, PMID: 38936799

[43] Barrett AJ. Fluorimetric assays for cathepsin B and cathepsin H with methylcoumarylamide substrates. Biochem J. (1980) 187:909–12. doi: 10.1042/bj1870909, PMID: 6897924 PMC1162479

[44] Morrison JF. Kinetics of the reversible inhibition of enzyme-catalysed reactions by tight-binding inhibitors. Biochim Biophys Acta. (1969) 185:269–86. doi: 10.1016/0005-2744(69)90420-3, PMID: 4980133

[45] Bizzarro B Barros MS Maciel C Gueroni DI Lino CN Campopiano J. Effects of *Aedes aegypti* salivary components on dendritic cell and lymphocyte biology. Parasit Vectors. (2013) 6:P. 329. doi: 10.1186/1756-3305-6-329, PMID: 24238038 PMC3843549

[46] Esteves E Bizzarro B Costa FB Ramírez-Hernández A Peti APF Cataneo AHD. *Amblyomma sculptum* salivary PGE_2_ modulates the dendritic cell-*Rickettsia rickettsii* interactions *in vitro* and *in vivo* . Front Immunol. (2019) And:P. 118. doi: 10.3389/fimmu.2019.00118, PMID: 30778355 PMC6369204

[47] Barros MS Gomes E Gueroni DI Ramos A Mirotti L Florsheim E. Exposure to *Aedes aegypti* bites induces A mixed-type allergic response following salivary antigens challenge in mice. PloS One. (2016) 11:E0155454. doi: 10.1371/journal.pone.0155454, PMID: 27203689 PMC4874626

[48] Costa GCA Ribeiro ICT Melo-Junior OG Gontijo NF Sant’Anna MRV Pereira MH. Salivary protease inhibitors as potential anti-tick vaccines. Front Immunol. (2020) 11:P. 611104. doi: 10.3389/fimmu.2020.611104, PMID: 33633731 PMC7901972

[49] Lara PG Esteves E Sales-Campos H Assis JB Henrique MO Barros MS. Aemope-1, a novel salivary peptide from *Aedes aegypti*, selectively modulates activation of murine macrophages and ameliorates experimental colitis. Front Immunol. (2021) 12:P. 681671. doi: 10.3389/fimmu.2021.68167, PMID: 34349757 PMC8327214

[50] Esteves E Maruyama SR Kawahara R Fujita A Martins LA Righi AA. Analysis of the salivary gland transcriptome of unfed and partially fed *Amblyomma sculptum* ticks and descriptive proteome of the saliva. Front Cell Infect Microbiol. (2017) 7:P. 476. doi: 10.3389/fcimb.2017.00476, PMID: 29209593 PMC5702332

[51] Lu S Soares TS Vaz Junior IS Lovato DV Tanaka AS. Rmcystatin3, A cysteine protease inhibitor from *Rhipicephalus microplus* hemocytes involved in immune response. Biochimie. (2014) 106:17–23. doi: 10.1016/j.biochi.2014.07.012, PMID: 25064361

[52] Cardoso THS Lu S Gonzalez BRG Torquato RJS Tanaka AS. Characterization of A novel cystatin type 2 from *Rhipicephalus microplus* midgut. Biochimie. (2017) 140:117–121. doi: 10.1016/j.biochi.2017.07.005, PMID: 28735872

[53] Lu S da Rocha LA Torquato RJS da Silva Vaz Junior I Florin-Christensen M Tanaka AS. A novel type 1 cystatin involved in the regulation of *Rhipicephalus microplus* midgut cysteine proteases. Ticks Tick Borne Dis. (2020) 11:P. 101374. doi: 10.1016/j.ttbdis.2020.101374, PMID: 32008997

[54] Grunclová L Horn M Vancová M Sojka D Franta Z Mareš M. Two secreted cystatins of the soft tick *Ornithodoros moubata*: differential expression pattern and inhibitory specificity. Biol Chem. (2006) 387:1635–44. doi: 10.1515/BC.2006.204, PMID: 17132111

[55] Yamaji K Tsuji N Miyoshi T Hatta T Alim MA Anisuzzaman K. Hlcyst-1 and Hlcyst-2 are potential inhibitors of hlcpl-A in the midgut of the ixodid tick *Haemaphysalis longicornis* . J Vet Med Sci. (2010) 72:599–604. doi: 10.1292/jvms.09-0561, PMID: 20103991

[56] Bode W Engh R Musil D Thiele U Huber R Karshikov A. The 2.0 A X-ray crystal structure of chicken egg white cystatin and its possible mode of interaction with cysteine proteinases. EMBO J. (1988) 7:2593–9. doi: 10.1002/j.1460-2075.1988.tb03109.x, PMID: 3191914 PMC457133

[57] Abrahamson M Alvarez-Fernandez M Nathanson CM. Cystatins. Biochem Soc Symp. (2003) 70:179–99. doi: 10.1042/bss0700179, PMID: 14587292

[58] Bode W Engh R Musil D Laber B Stubbs M Huber R. Mechanism of interaction of cysteine proteinases and their protein inhibitors as compared to the serine proteinase-inhibitor interaction. Biol Chem Hoppe Seyler. (1990) 371 Suppl:111–8., PMID: 2205234

[59] Sá-Nunes A Bafica A Antonelli LR Choi EY Francischetti IM Andersen JF. The immunomodulatory action of Sialostatin L on dendritic cells reveals its potential to interfere with autoimmunity. J Immunol. (2009) 182:7422–9. doi: 10.4049/jimmunol.0900075, PMID: 19494265 PMC2694955

[60] Zavašnik-Bergant T Vidmar R Sekirnik A Fonović M Salát J Grunclová L. Salivary tick cystatin Omc2 targets lysosomal cathepsins S and C in human dendritic cells. Front Cell Infect Microbiol. (2017) 7:P. 288. doi: 10.3389/fcimb.2017.00288, PMID: 28713775 PMC5492865

[61] Sá-Nunes A Oliveira CJF. Dendritic cells as A disputed fortress on the tick-host battlefield. Trends Parasitol. (2021) 37:340–354. doi: 10.1016/j.pt.2020.11.004, PMID: 33303363

[62] Hsing LC Rudensky AY. The lysosomal cysteine proteases in mhc class ii antigen presentation. Immunol Rev. (2005) 207:229–41. doi: 10.1111/j.0105-2896.2005.00310.x, PMID: 16181340

[63] Roche PA Furuta K. The ins and outs of MHC class ii-mediated antigen processing and presentation. Nat Rev Immunol. (2015) 15:203–16. doi: 10.1038/nri3818, PMID: 25720354 PMC6314495

[64] Kotsyfakis M Anderson JM Andersen JF Calvo E Francischetti IM Mather TN. Cutting edge: immunity against A “Silent” Salivary antigen of the lyme vector *Ixodes scapularis* impairs its ability to feed. J Immunol. (2008) 181:5209–12. doi: 10.4049/jimmunol.181.8.5209, PMID: 18832673 PMC2562228

[65] Sá-Nunes A Bafica A Lucas DA Conrads TP Veenstra TD Andersen JF. Prostaglandin E2 is a major inhibitor of dendritic cell maturation and function in *Ixodes scapularis* saliva. J Immunol. (2007) 179:1497–505. doi: 10.4049/jimmunol.179.3.1497, PMID: 17641015

[66] Oliveira CJ Sá-Nunes A Francischetti IM Carregaro V Anatriello E Silva JS. Deconstructing tick saliva: non-protein molecules with potent immunomodulatory properties. J Biol Chem. (2011) 286:10960–9. doi: 10.1074/jbc.M110.205047, PMID: 21270122 PMC3064151

[67] Moore KW O’Garra A de Waal Malefyt R Vieira P Mosmann TR. Interleukin-10. Annu Rev Immunol. (1993) 11:165–90. doi: 10.1146/annurev.iy.11.040193.001121, PMID: 8386517

[68] Ouyang W Rutz S Crellin NK Valdez PA Hymowitz SG. Regulation and functions of the IL-10 family of cytokines in inflammation and disease. Annu Rev Immunol. (2011) 29:71–109. doi: 10.1146/annurev-immunol-031210-101312, PMID: 21166540

[69] Hartmann S Rutz S Crellin NK Valdez PA Hymowitz SG. A filarial cysteine protease inhibitor down-regulates T cell proliferation and enhances interleukin-10 production. Eur J Immunol. (1997) 27:2253–60. doi: 10.1002/eji.1830270920, PMID: 9341767

[70] Klotz C Ziegler T Figueiredo AS Rausch S Hepworth MR Obsivac NS. A helminth immunomodulator exploits host signaling events to regulate cytokine production in macrophages. PloS Pathog. (2011) 7:E1001248. doi: 10.1371/journal.ppat.1001248, PMID: 21253577 PMC3017123

[71] Schuijs MJ Hartmann S Selkirk ME Roberts LB Openshaw PJ Schnoeller C. The helminth-derived immunomodulator avcystatin reduces virus enhanced inflammation by induction of regulatory IL-10+ T cells. PloS One. (2016) 11:E0161885. doi: 10.1371/journal.pone.0161885, PMID: 27560829 PMC4999285

[72] Schönemeyer A Lucius R Sonnenburg B Brattig N Sabat R Schilling K. Modulation of human T cell responses and macrophage functions by onchocystatin, A secreted protein of the filarial nematode *Onchocerca volvulus* . J Immunol. (2001) 167:3207–15. doi: 10.4049/jimmunol.167.6.3207, PMID: 11544307

[73] Jang SW Cho MK Park MK Kang SA Na BK Ahn SC. Parasitic helminth cystatin inhibits dss-induced intestinal inflammation via IL-10(+)F4/80(+) macrophage recruitment. Korean J Parasitol. (2011) 49:245–54. doi: 10.3347/kjp.2011.49.3.245, PMID: 22072824 PMC3210841

[74] Xie H Wu L Chen X Gao S Li H Yuan Y. Cystatin alleviates sepsis through activating regulatory macrophages. Front Cell Infect Microbiol. (2021) 11:P. 617461. doi: 10.3389/fcimb.2021.617461, PMID: 33718268 PMC7943722

[75] Li H Qiu D Yuan Y Wang X Wu F Yang H. *Trichinella spiralis* cystatin alleviates polymicrobial sepsis through activating regulatory macrophages. Int Immunopharmacol. (2022) 109:P. 108907. doi: 10.1016/j.intimp.2022.108907, PMID: 35691271

[76] Wu H Jmel MA Chai J Tian M Xu X Hui Y. Tick cysteine protease inhibitors suppress immune responses in mannan-induced psoriasis-like inflammation. Front Immunol. (2024) 15:P. 1344878. doi: 10.3389/fimmu.2024.1344878, PMID: 38444844 PMC10912570

[77] Tatchell RJ Moorhouse DE. Neutrophils: their role in the formation of a tick feeding lesion. Science. (1970) 167:1002–3. doi: 10.1126/science.167.3920.1002, PMID: 5411168

[78] Brown SJ Knapp FW. *Amblyomma americanum*: sequential histological analysis of larval and nymphal feeding sites on Guinea pigs. Exp Parasitol. (1980) 49:188–205. doi: 10.1016/0014-4894(80)90116-2, PMID: 7364007

[79] Brown SJ. Antibody- and cell-mediated immune resistance by Guinea pigs to adult *Amblyomma americanum* ticks. Am J Trop Med Hyg. (1982) 31:1285–90. doi: 10.4269/ajtmh.1982.31.1285, PMID: 7149113

[80] Brown SJ Worms MJ Askenase PW. *Rhipicaphalus appendiculatus*: larval feeding sites in Guinea pigs actively sensitized and receiving immune serum. Exp Parasitol. (1983) 55:111–20. doi: 10.1016/0014-4894(83)90004-8, PMID: 6822283

[81] Chudzinski-Tavassi AM De-Sá-Júnior PL Simons SM Maria DA de Souza Ventura J Batista IF. A new tick kunitz type inhibitor, Amblyomin-X, induces tumor cell death by modulating genes related to the cell cycle and targeting the ubiquitin-proteasome system. Toxicon. (2010) 56:1145–54. doi: 10.1016/j.toxicon.2010.04.019, PMID: 20570593

[82] Maria DA Will SE Bosch AL Souza JV Sciani JM Goldfeder MB. Preclinical evaluation of Amblyomin-X, A Kunitz-type protease inhibitor with antitumor activity. Toxicol Rep. (2019) 6:51–63. doi: 10.1016/j.toxrep.2018.11.014, PMID: 30581760 PMC6298944

[83] Franck C Foster SR Johansen-Leete J Chowdhury S Cielesh M Bhusal RP. Semisynthesis of an evasin from tick saliva reveals a critical role of tyrosine sulfation for chemokine binding and inhibition. Proc Natl Acad Sci U.S.A. (2020) 117:12657–12664. doi: 10.1073/pnas.2000605117, PMID: 32461364 PMC7293604

