# Supplementary material for: Amblyostatin-1, the first salivary cystatin with host immunomodulatory and anti-inflammatory properties from the Neotropical tick Amblyomma sculptum, vector of Brazilian spotted fever
Source: Front Immunol. 2025 Jul 17;16:1585703. doi: 10.3389/fimmu.2025.1585703 (PMC12310653; doi:10.3389/fimmu.2025.1585703)
Supplement: Supplementary file 1 [file DataSheet1.pdf]

Supplementary Figure 1

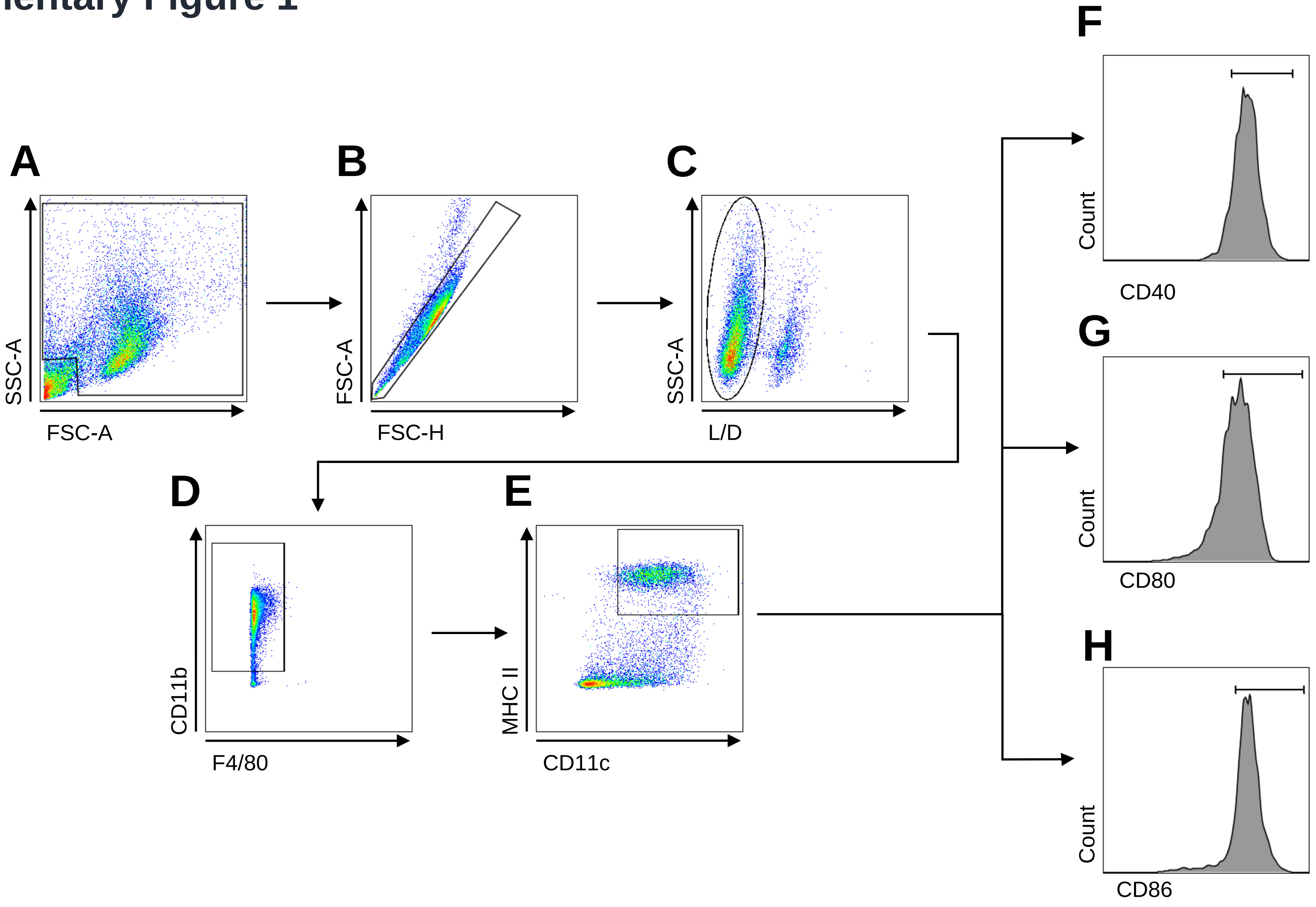

Supplementary Figure 1. Gating strategy for the analysis of accessory/costimulatory molecules expressed by DCs. (A) Small-sized events and debris exclusion based on forward scatter (FSC) and side scatter (SSC) parameters. (B) Doublets exclusion using the area (FSC-A) and height (FSC-H) parameters. (C) Exclusion of dead cells, identified as Live/Dead-positive events. (D and E) Selection of $CD11b^{+}F4/80^{-}CD11c^{+}MHC^{high}$ cells (considered DCs). From the DC population, the percentage of $CD40^{+}$ (F), $CD80^{+}$ (G), and $CD86^{+}$ (H) cells was assessed and the median fluorescence intensity (MFI) calculated.

Supplementary Figure 2

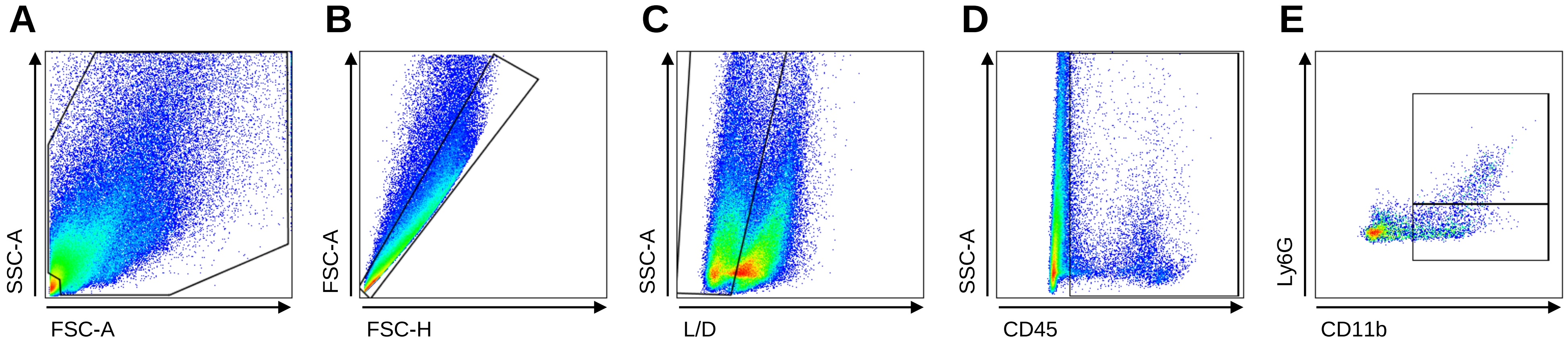

Supplementary Figure 2. Gating strategy for the analysis of cell infiltrate in carrageenan-induced paw edema. (A) Small-sized events and debris exclusion based on FSC and SSC parameters. (B) Exclusion of doublets using FSC-A and FSC-H parameters. (C) Exclusion of dead cells, identified as exclusion Live/Dead-positive events. (D and E) Selection of CD45+CD11b^{+}Ly6G^{+} cells (neutrophils) and CD45+CD11b^{+}Ly6G^{-} cells (other myeloid cells).

Supplementary Figure 3

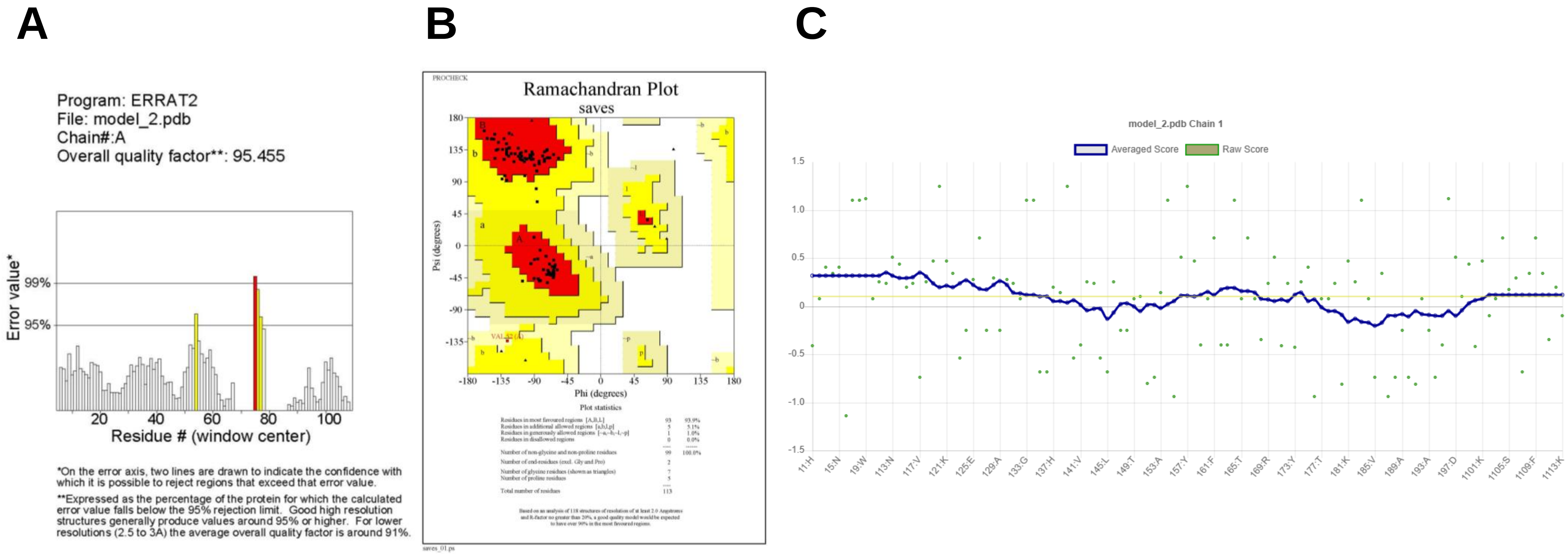

Supplementary Figure 3. SAVES validation output. **(A)** ERRAT2 analysis showing an overall quality factor of 95.455, indicating a high-quality model. **(B)** Ramachandran plot with 93.9% of residues in the most favored regions, supporting good stereochemical quality. **(C)** Verify3D results indicating that 54.87% of the residues have an averaged 3D-1D score ≥ 0.1, suggesting moderate compatibility between the 3D model and its amino acid sequence.

Supplementary Figure 4

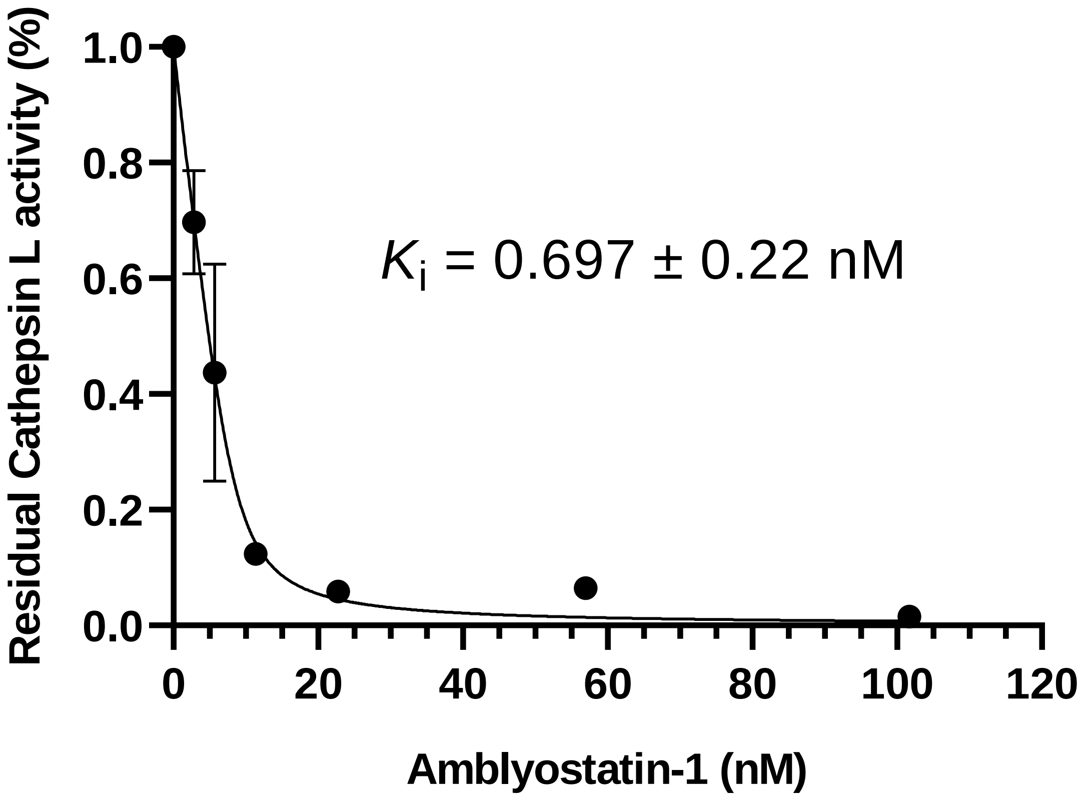

Supplementary Figure 4. Residual cathepsin L activity in the presence of Amblyostatin-1. Active cathepsin L (6 nM) was incubated with varying concentrations of Amblyostatin-1 followed by addition of the fluorogenic substrate *Z*-Phe-Arg-AMC Fluorescence readings were taken at 30 °C over a 15 minutes period, and enzyme activity was estimated by their Vmax. Residual activity was calculated as Vmax of enzyme activity in the presence of the inhibitor divided by the Vmax of the control enzyme (without inhibitor). The dissociation constant (K_i) was calculated through nonlinear regression analysis using the Morrison equation for tight-binding inhibition.

Supplementary Table 1

Supplementary Table 1. Tested human cathepsins with its respective substrates and reaction buffers.

Enzyme	Final enzyme concentration	Substrate	Final substrate concentration	Reaction butter
Cathepsin L	250 nM	Z-LR-AMC	250 µM	100 mM Na-acetate, 100 mM NaCl, 1 mM EDTA, 0.01 % Triton X-100, 100 µg/ml cysteine, pH 5.5
Cathepsin B	85.5 nM	Z-LR-AMC	250 µM	
Cathepsin H	286 nM	Z-LR-AMC	250 µM	
Cathepsin S	169 nM	VVR-AMC	250 µM	
Cathepsin C	50 nM	H-GR-AMC	250 µM	50 mM Na-acetate, 50 mM NaCl, 5 mM DTT, pH 5.5
